# Association genetics in *Solanum tuberosum *provides new insights into potato tuber bruising and enzymatic tissue discoloration

**DOI:** 10.1186/1471-2164-12-7

**Published:** 2011-01-05

**Authors:** Claude Urbany, Benjamin Stich, Lysann Schmidt, Ludwig Simon, Hergen Berding, Holger Junghans, Karl-Heinz Niehoff, Alexander Braun, Eckhard Tacke, Hans-Rheinhardt Hofferbert, Jens Lübeck, Josef Strahwald, Christiane Gebhardt

**Affiliations:** 1 Max Planck Institute for Plant Breeding Research, 50829 Cologne; Germany; 2 Bavaria Saat BGB GmbH, 86529 Schrobenhausen; Germany; 3 Saatzucht Berding, 26345 Bockhorn-Petersgroden; Germany; 4 NORIKA, 18190 Groß Lüsewitz; Germany; 5 Dr. K.-H. Niehoff, Gut Bütow, 17209 Bütow; Germany; 6 Böhm-Nordkartoffel Agrarproduktion OHG, 84085 Langquaid; Germany; 7 BIOPLANT GmbH, 29547 Ebstorf; Germany; 8 Böhm-Nordkartoffel Agrarproduktion GbR, 29574 Ebstorf; Germany; 9 Saka-Pflanzenzucht G.b.R., 24340 Windeby; Germany

## Abstract

**Background:**

Most agronomic plant traits result from complex molecular networks involving multiple genes and from environmental factors. One such trait is the enzymatic discoloration of fruit and tuber tissues initiated by mechanical impact (bruising). Tuber susceptibility to bruising is a complex trait of the cultivated potato (*Solanum tuberosum*) that is crucial for crop quality. As phenotypic evaluation of bruising is cumbersome, the application of diagnostic molecular markers would empower the selection of low bruising potato varieties. The genetic factors and molecular networks underlying enzymatic tissue discoloration are sparsely known. Hitherto there is no association study dealing with tuber bruising and diagnostic markers for enzymatic discoloration are rare.

**Results:**

The natural genetic diversity for bruising susceptibility was evaluated in elite middle European potato germplasm in order to elucidate its molecular basis. Association genetics using a candidate gene approach identified allelic variants in genes that function in tuber bruising and enzymatic browning. Two hundred and five tetraploid potato varieties and breeding clones related by descent were evaluated for two years in six environments for tuber bruising susceptibility, specific gravity, yield, shape and plant maturity. Correlations were found between different traits. In total 362 polymorphic DNA fragments, derived from 33 candidate genes and 29 SSR loci, were scored in the population and tested for association with the traits using a mixed model approach, which takes into account population structure and kinship. Twenty one highly significant (p < 0.001) and robust marker-trait associations were identified.

**Conclusions:**

The observed trait correlations and associated marker fragments provide new insight in the molecular basis of bruising susceptibility and its natural variation. The markers diagnostic for increased or decreased bruising susceptibility will facilitate the combination of superior alleles in breeding programs. In addition, this study presents novel candidates that might control enzymatic tissue discoloration and tuber bruising. Their validation and characterization will increase the knowledge about the underlying biological processes.

## Background

Enzymatic browning is a phenomenon frequently observed in fruits and vegetables upon physical damage. The tissue discoloration results from the oxidation of endogenous phenolic compounds, which is catalyzed by oxidoreductases, notably polyphenol oxidases (PPOs) or tyrosinases in the presence of oxygen [1-8]. Besides the unappealing tissue pigmentation, enzymatic browning affects nutritional properties, flavour and texture of food and feed during storage or processing and is therefore detrimental to food quality. Mechanical impact during harvest, transport and storage of potato tubers initiates the development of an internal tissue discoloration ('blackspot bruising'). Blackspot bruising leads to the rejection of the crop by consumers, the retail and the processing industry, which results in considerable economic losses. 'After cooking darkening' is another problematic consequence of enzymatic browning.

**Figure 1:**
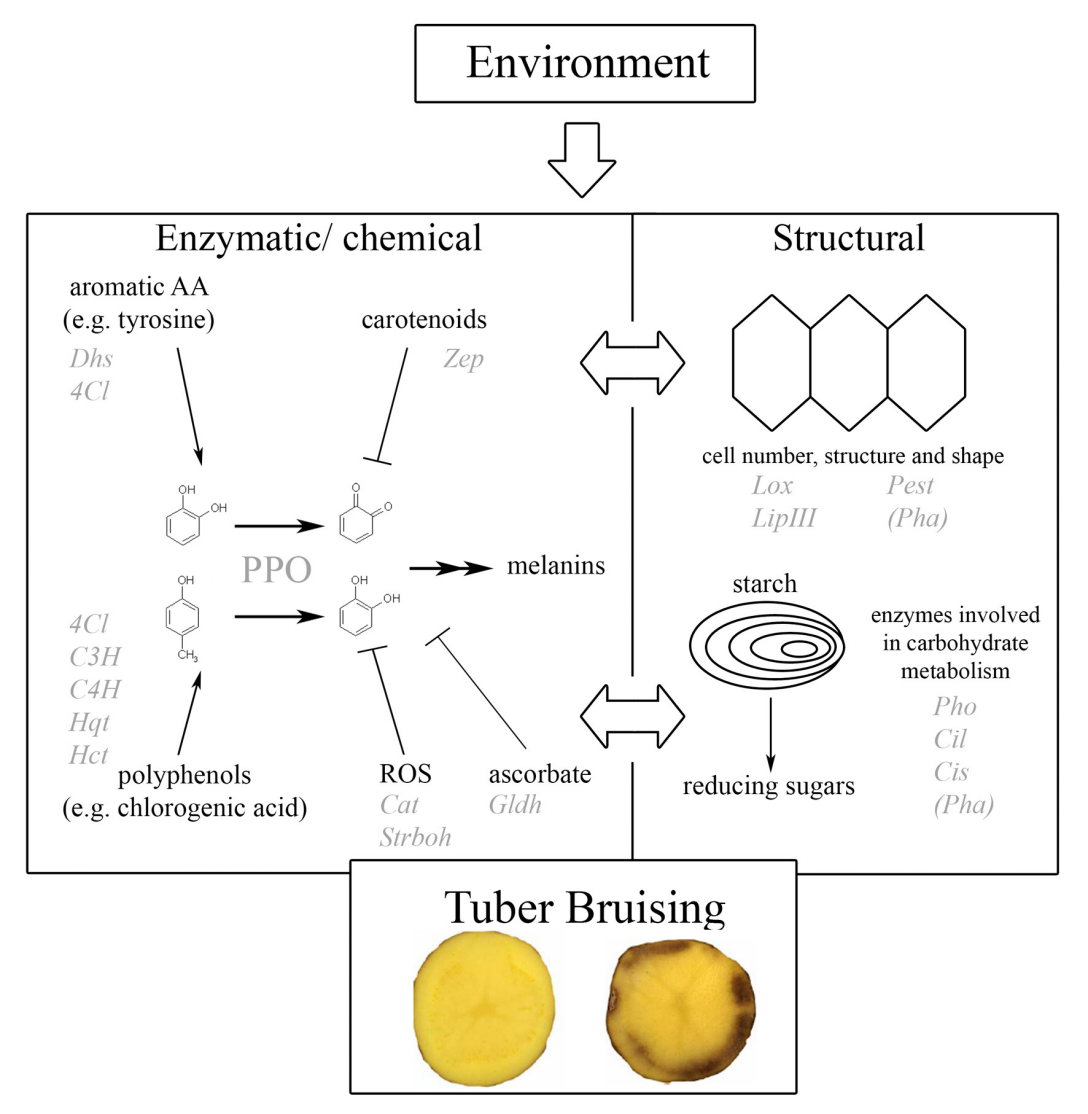
**Simplified metabolic scheme of potato tuber bruising and the candidate genes used in the association experiment**. Shown are the enzymatic/chemical, structural and environmental components and the involved genes with their corresponding abbreviations. The abbreviations are: AA = Amino acid; ROS = reactive oxygen species PPO = Polyphenoloxidase, 4Cl = 4-coumarate:CoA ligase, C3H = p-coumarate 3-hydroxylase, C4H = Cinnamic acid 4-hydroxylase; Cil = ATP citrate lyase, Cis = Mitochondrial citrate synthase, Cat = Catalase, Dhs = 3-deoxy-7-phosphoheptulonate synthase, Gldh = L-galactono-1,4-lactone dehydrogenase, Hct = Hydroxycinnamoyl transferase, Hqt = Hydroxycinnamoyl CoA quinate transferase, LipIII = Triacylglycerol lipase III, Lox = Lipoxygenase, Pest = pectin methyl esterase, Pha = plasma memebrane ATPase; proton pump, Pho = α-glucan phosphorylase L-type, Strboh = *Solanum tuberosum *respiratory burst oxidase homologue, Zep = zeaxanthin epoxidase.

When cells are damaged, phenolic compounds present in the vacuoles are oxidized to quinones by PPOs located in the plastids (chloroplasts and amyloplasts). The quinones polymerize non-enzymatically further into dark pigments (melanins), attributing a dark gray to black appearance to the normally white to yellowish tissue [9,10] (Figure 1). Bruising symptoms emerge 3 to 24 hours after the impact and the damaged tissue area is only seen after removal of the tuber skin. Previous studies report that the discoloration reaction strongly depends on PPO enzyme activity [11-14]. Potato PPOs are encoded by a small gene family of at least six members, namely *POTP1 *and *POTP2 *[15], *POT32, POT33, POT41*, and *POT72 *[16], which show tissue specific expression. In contrast to *POTP1 *and *POTP2*, which are mainly expressed in leaves and flowers [16], *POT32 *is considered to be the major form expressed in tubers besides *POT33 *and *POT72*. Silencing PPO gene expression resulted in transgenic potato lines that exhibited a significantly reduced discoloration reaction [17-19]. This observation further points to the important role of PPO enzyme activity for bruising. In addition, it has been shown that molecular markers derived from the *POT32 *sequence co-localise with a quantitative trait locus (QTL) for enzymatic discoloration on potato chromosome VIII in a diploid potato mapping population [20]. Taken together, functional and genetic studies suggest *PPOs *as major candidate genes for influencing tissue discoloration and tuber bruising. The role of the cellular levels of the PPO substrates is less clear. Contrasting findings do not allow a clear statement on the impact on discoloration and tuber bruising of chlorogenic acid contents, the main phenolic compound in potato tubers, of its precursor tyrosine and the partitioning of tyrosine between tuber protein and the free amino acid pool [10,21,22]. In addition to PPO and its substrates, reducing agents such as ascorbate and carotenoids as well as redox homeostasis influence tuber bruising [23,24]; Figure 1. Besides the enzymatic component, structural factors like cellular membrane stability, cell number and architecture also affect tuber bruising [9,25] (Figure 1). The latter characters not only influence tuber bruising but are also involved in other tuber traits like tuber shape, starch and sugar content of tubers, and tuber yield [26,27]. Starch accounts for 10-25% of the tuber fresh weight and is the major storage compound of potato tubers [28]. The amount and steric properties of starch granules influence tuber starch content, which correlates with specific gravity [29] and thereby affect cellular stability and tuber bruising.

The susceptibility of potato tubers to blackspot bruising is a complex trait, which depends on multiple genetic factors, developmental stage and environment [9]. Potato breeding programs aim at the selection of cultivars with improved genetic resistance to bruising as an important quality trait. However, the phenotypic evaluation of bruising susceptibility is difficult. It requires large amounts of tubers, which become only available after several years of vegetative multiplication. Multi-year and -location trials are needed to separate genetic variability from the environmental one. This prevents the selection of low bruising cultivars early in the breeding cycle. An additional problem is the correlation between blackspot bruising and specific gravity, which makes the selection of superior alleles for resistance to blackspot bruising independent from specific gravity difficult. Identifying the molecular basis of the genetic variation of bruising susceptibility will therefore facilitate the early selection of low bruising cultivars by means of molecular markers. Such markers can be used to diagnose and select for or against superior or inferior alleles of the genes themselves that control bruising in parents and progeny of breeding programs. Besides application in breeding, knowledge of which and how gene variants influence the bruising phenotype will contribute to understanding the complex molecular and cellular networks involved in enzymatic browning in plants.

The identification of quantitative trait loci (QTL) underlying complex phenotypic traits became feasible with the introduction of DNA-based markers [30]. Since then, a number of genes and their allelic variants underlying plant QTL have been identified by positional cloning [reviewed in [31]]. Positional QTL cloning is a laborious and time-intensive process, which is feasible in inbreeding species such as *Arabidopsis *and rice but is severely handicapped in polyploid, non-inbred species such as potato [32]. Moreover, the bruising phenotype itself is rather prohibitive for positional cloning due to the difficulties of the phenotypic evaluation as outlined above. An alternative to positional QTL cloning is the candidate gene approach, which is based on the knowledge of a gene's function in controlling a character of interest on the one hand, and genetic co-localization of a functional candidate gene with QTL for the character of interest on the other [33]. The investigation of DNA variation at loci fulfilling these criteria in natural populations of individuals related by descent [34] can lead to the detection of associations with positive or negative character values [35-38]. Finding such associations indicates that DNA variation, either at the candidate locus itself or at a linked locus is causal for the phenotypic variation. The extent of linkage disequilibrium (LD) present in the population under study determines the amount of indirect associations due to physical linkage. Validation of a direct association between a candidate gene allele and a QTL effect can be achieved by quantitative complementation analysis [38]. Whether the association is direct or indirect, association mapping can produce diagnostic markers for superior alleles of genes influencing phenotypic variation, thereby empowering plant breeding.

To dissect the molecular basis of tuber bruising susceptibility and to identify diagnostic markers for "precision breeding", we conducted an association mapping study in a population of tetraploid genotypes used for potato variety development. In addition to bruising, the population was phenotyped for specific gravity, yield, tuber shape and plant maturity, in order to determine the correlation of these traits with bruising susceptibility. We genotyped the population with a series of DNA-based markers developed from candidate gene sequences. Candidate genes (Figure 1) were selected from the scientific literature and de novo from comparative proteomics of bruising resistant versus bruising susceptible cultivars (Urbany et al., in preparation). Highly significant marker-trait associations were detected for bruising susceptibility and other traits, which are discussed with respect to the mechanisms underlying natural variation of enzymatic discoloration and tuber bruising.

## Results

### Phenotypic analysis

**Figure 2:**
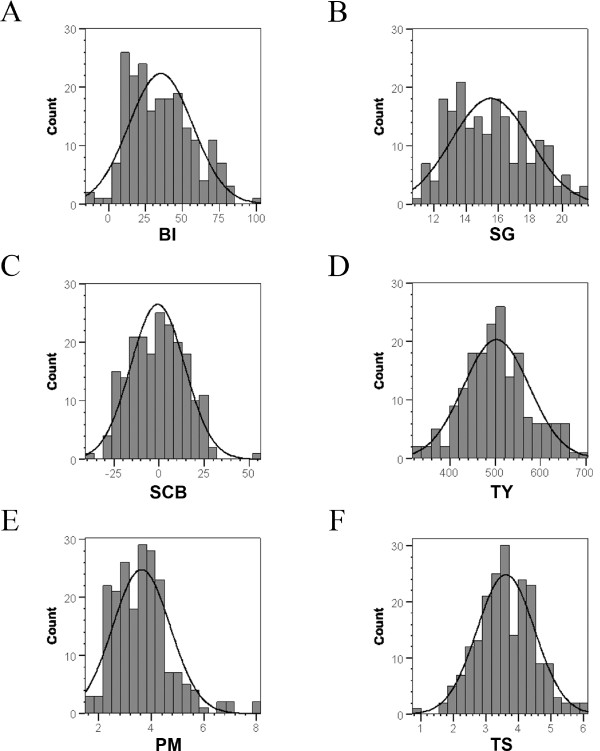
**Phenotypic distribution of adjusted means of tuber quality traits**. The histograms display the number of genotypes (counts) that are present in a specific phenotypic class for the given trait. (A) Bruising index (BI) from 0 to 100 was calculated as described in materials and methods. (B) Specific gravity (SG) is displayed as percent fresh weight. (C) The values for starch corrected bruising (SCB) were derived from BI and SG as described in materials and methods (D) Tuber yield (TY) is shown as decitons per hectar. (E) Plant maturity (PM) and (F) tuber shape (TS) are given as scores between 1 (very early, circular tuber shape) and 9 (very late, oblong tuber shape). Histograms and the normal distribution curve were generated using SPSS software (SPSS Inc; Chicago, Illinois 60606 USA).

**Figure 3:**
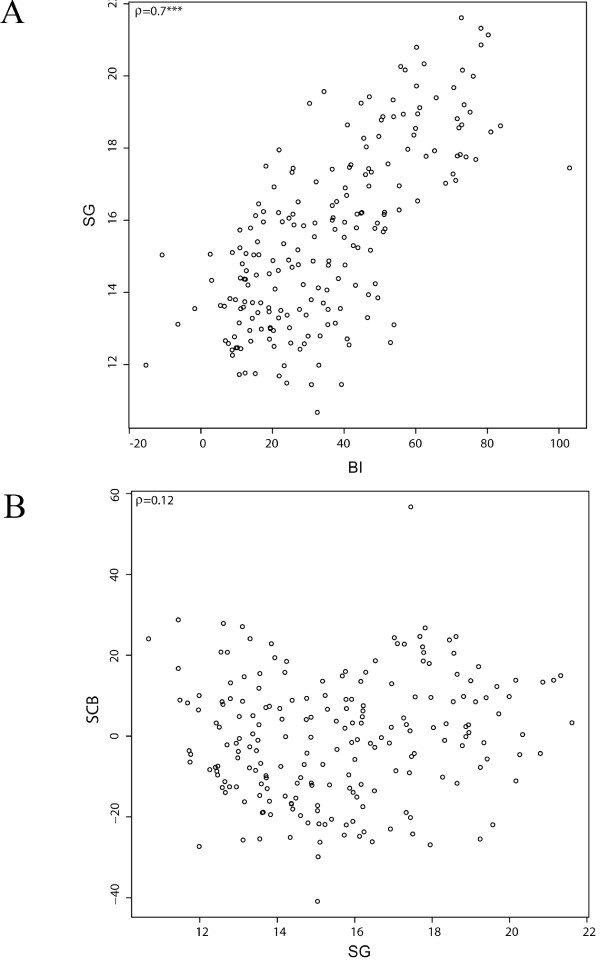
**Correlation plots between specific gravity and bruising index (A) and starch corrected bruising (B)**. Genotypes were plotted according to their adjusted entry means for the respective trait. ρ is the correlation coefficient. Significant trait correlation is indicated by ***. Bruising index (BI), specific gravity (SG), starch corrected bruising (SCB).

Balanced phenotypic data from two years were obtained for 205 genotypes (85 varieties evaluated at six locations, and 6 × 20 breeding clones evaluated at one location each, (Additional file 1), except for yield (NE and SA data sets missing) and maturity (NE data set missing). The phenotypic and genotypic data of the six data sets EU, SA, NO, BS, NE and BE, corresponding to location and breeder, were combined and constituted the data set ALL, which was used for the association analysis. Heritability (*H*^2^) was high for all traits except yield. Apart from the NE data set, bruising index (BI) as well as starch corrected bruising (SCB) showed a high overall heritability. Specific gravity (SG) was characterized by high *H*^2 ^values in every single data set. Low *H*^2 ^values were identified for tuber shape (TS) and plant maturity (PM) in the NO data set and in all single data sets concerning tuber yield (TY) (Table 1). The phenotypic distributions (adjusted entry means) of BI, SG, SCB, TY TS and PM in the ALL population are given in Figure 2. The ALL population showed a trend to BI below 30 points and SG between 12 and 16 percent (Figure 2A, B). In addition, genotypes were characterized by a mid plant maturity of about four or lower on the scale ranging from zero to nine (Figure. 2E). Correlation analyses of the phenotypic data pinpointed to a highly significant, positive correlation between BI and SG (Figure 3A). Accordingly, the majority of the genotypes highly susceptible to bruising had a specific gravity of 15% and more (Figure 3A). SCB did not correlate with SG, and genotypes with positive SCB scores, indicating bruising susceptibility, were distributed evenly over all SG classes (Figure 3B). Significant correlations were also observed between some other traits (Table 2). SCB clearly correlated positively with BI and negatively with TS. Genotypes highly susceptible to bruising were characterized by elevated SCB values and a trend of circular to ovoid tuber shape that also correlated with increasing SG. PM correlated slightly but significantly with BI as well as with SG. Later maturing genotypes showed on average a higher specific gravity and elevated bruising susceptibility. A very weak positive correlation between yield and tuber shape and a negative one between yield and SG were also identified.

**Table 1:** Heritability (H2) of bruising index (BI), specific gravity (SG), starch corrected bruising (SCB), tuber yield (TY), plant maturity (PM) and tuber shape (TS)

Data set	BI	SG	SCB	TY	PM	TS
**BS**	0.874	0.931	0.684	0.540	0.738	0.841
**NO**	0.904	0.902	0.905	0.622	0.590	0.499
**EU**	0.928	0.971	0.880	0.641	0.845	0.817
**BE**	0.783	0.957	0.632	0.262	0.999	0.640
**SA**	0.914	0.966	0.812	NA	0.938	0.928
**NE**	0.488	0.804	0.406	NA	NA	0.798
**ALL**	0.864	0.925	0.772	0.375	0.831	0.819

NA = Not available; BS = Bavaria Saat; NO = Norika; EU = Europlant; BE = Berding; SA = Saka; NE = Niehoff; ALL = combined data set

Values were obtained using collected data in a population of 205 tetraploid potato genotypes in a two year trial at multiple environments.

**Table 2:** Correlation matrix of bruising index (BI), specific gravity (SG), starch corrected bruising (SCB), tuber yield (TY), plant maturity (PM) and tuber shape (TS)

	BI	SG	SCB	TY	PM
**SG**	0.7***				
**SCB**	0.78***	ns			
**TY**	ns	-0.18*	ns		
**PM**	0.35***	0.46***	ns	0.25***	
**TS**	-0.47***	-0.44***	-0.28***	0.17*	ns

* significant at α = 0.05; *** significant at α = 0.001, ns = not significant

### Association analysis

**Figure 4:**
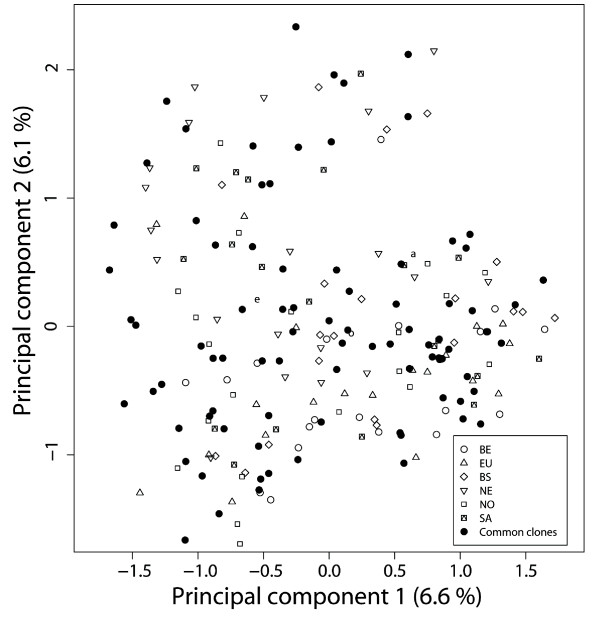
**Principal coordinate analysis of 205 tetraploid potato genotypes**. Principal coordinate analysis was performed using the SSR genotyping data. The common varieties are represented with black dots. Breeding clones are referred to by the corresponding breeder abreviation. Berding (BE), Europlant (EU), Bavaria Saat (BS), Niehoff (NE), Norika (NO), SaKa (SA) and the corresponding symbol.

First, population structure was analysed by principal coordinate analysis using the genotypic data of 157 SSR alleles scored at 29 loci (between 1 and 9 alleles per locus) distributed on all twelve potato chromosomes. No distinct subgroups were observed (Figure 4). A total of 362 polymorphic DNA fragments derived from 33 candidate genes and 29 SSR loci were scored in the 205 individuals (Additional file 2 and 3). DNA polymorphisms were tested individually for association with all traits using a mixed model approach, which takes into account population structure and kinship, as well as by stepwise forward regression. Twenty one and eighty six marker-trait associations were found that were significant at P < 0.001 (Table 3; for exemplary separation panes see Additional file 4) and P < 0.01 (Additional file 2), respectively. At P < 0.001, 13 polymorphic fragments that originated from five candidate and two SSR loci were associated with tuber bruising (BI and/or SCB (Table 3)). Interestingly, markers derived from PPO isoforms influenced tuber bruising in opposite directions, either decreasing or increasing bruising susceptibility compared to the population mean. For example, the SSCP marker *POT32PS1-f *with a frequency of only ten percent in the ALL population, decreased BI and SCB by 14.4 and 12.4 points, respectively. In contrast, the *POLOXA *marker with a frequency of 35 percent increased BI and SCB by 11.2 and 7.8 points, respectively (Additional file. 2). Markers from the starch phosphorylase *PHO1A *were all associated with increased bruising susceptibility, whereas markers from lipase class III, 4-coumarate CoA ligase and HQT on average lowered bruising (Table 3). In addition, the SSR alleles *StI024-e *and *StI013-a *showed association with BI and SG but not with SCB. In contrast to the highly frequent *StI024-e *allele (90% presence in the population), which decreased bruising susceptibility and specific gravity (Table 3), the *StI013-a *allele increased trait values when compared to population mean scores.

**Table 3:** Marker trait associations in the ALL population (P < 0.001)

**Trait**^**1**^	Gene	Metabolism	Marker allele	Chromosome	Allele Effect	p value	**Fr. (%)**^**2**^
**BI**	Starch phosphorylase L-type	Starch	*PHO1A-b*	III	**^4^**↑ 17.8 ± 4	1.41·10^-5^	12.4
			*PHO1A-c*	III	↑ 13.3 ± 3.3	6.3·10^-5^	20.8
			*PHO1A-a*	III	↑ 20.6 ± 6	0.00070	7.1
	Lipase class III	Lipids	*LIPIII-27-1h*	II	↓ 15.8 ± 3.8	4.6·10^-5^	80.4
	PPO isoform potpoloxA	Polyphenols	*POLOXA*	VIII	↑ 11.2 ± 3.1	0.00033	35.5
	4-coumarate CoA Ligase	Phenylpropanoids	*4CL-1b*	III	↓ 10.6 ± 2.7	0.00016	59.1
			*4CL-2c*	III	↓ 9.5 ± 2.8	0.00091	49.3
	non coding SSR	-	*StI024-e*	II	↓ 17.9 ± 5.1	0.00051	90.2
	non coding SSR	-	*StI013-a*	III	↑ 11.1 ± 3.2	0.00058	44.4

**SG**	Starch phosphorylase L-type	Starch	*PHO1B-1b*	V	↓ 1.35 ± 0.37	0.00036	30.6
			*PHO1B-1a*	V	↓ 1.20 ± 0.34	0.00051	63.1
	Starch phosphorylase L-type	Starch	*PHO1A-b*	III	↑ 1.60 ± 0.46	0.00070	12.4
	non coding SSR	-	*StI024-e*	II	↓ 2.06 ± 0.58	0.00047	90.2
	non coding SSR	-	*StI013-a*	III	↑ 1.36 ± 0.36	0.00023	44.4
	non coding SSR	-	*SSR327-a*	VIII	↑ 1.80 ± 0.48	0.00026	17.7

**SCB**	Lipase class III	Lipids	*LIPIII-27-1h*	II	↓ 10.3 ± 2.8	0.00028	80.4
	Hydroxycinnamoyl quinate CoA transferase	Polyphenols	*HQT-c*	VII	↓ 13.0 ± 3.3	0.00023	45.9
	PPO isoform pot32	Polyphenols	*POT32PS1-Hpy-f*	VIII	↓ 12.4 ± 3.4	0.00039	10.2
	PPO isoform potpoloxA	Polyphenols	*POLOXA*	VIII	↑ 7.8 ± 2.3	0.00064	35.5

**TY**	L-galactono-1,4-lactone dehydrogenase	Ascorbate synthesis	*GLDH-h*	- **^3^**	↓ 55.7 ± 14.2	0.00012	81.3
	Hydroxycinnamoyl quinate CoA transferase	Polyphenols	*HQT-f*	VII	↑ 35.9 ± 10.6	0.00084	54.7

**^1 ^**BI: bruising index; SG: specific gravity; SCB: starch corrected bruising; TY: tuber yield.

**^2 ^**Frequency of presence of the marker fragment in the ALL population.

**^3 ^**Map position not known.

**^4 ^**Direction of effect either positive (**↑**) or negative (**↓**).

The candidate gene markers that showed highly significant association with SG were all derived from the starch phosphorylase genes *PHO1A *and *PHO1B *(alternative names *Stp23 *and *StpL*, respectively [35]). The *PHO1A-b *fragment increased, whereas the *PHO1B-1a *and *PHO1B-1b *fragments decreased specific gravity. In general, markers that were associated with both BI and SG showed a concerted allele effect, either increasing or decreasing bruising susceptibility and specific gravity (Table 3 and 4, Additional file 2). This corresponds to the phenotypic correlation observed between these traits. All *PHO1B *markers associated with both BI and SG did not show association with SCB. Markers specifically associated with tuber bruising (BI and/or SCB), independent from specific gravity, originated from the candidate genes *PPO *(*POLOXA, POT32PS1-f*), lipase class III (*LIPIII-27-1h*), hydroxycinnamoyl CoA quinate transferase (*HQT-c*), starch phosphorylase L-type (*PHO1A-a*) and the SSR locus *M20 *(*M20-b*) (Table 3 and 4, Additional file 2). The SSR locus *M20 *is located in a cluster of protease inhibitor genes on potato chromosome III [39]. Compared with the traits BI and SG, a lower number of markers were associated with tuber yield, shape and plant maturity. For TS and PM, no markers associated at P < 0.001 were identified. The markers associated at P < 0.01 are included in Additional file 2. Seven markers that were associated with BI, SG and/or SCB were also associated with TS (Table 4). In all cases, the direction of the effect on TS was opposite to the effect on BI, SG and SCB, in concordance with the correlation of round tuber shape with increased bruising susceptibility and specific gravity. Two unique and highly significant associations were found for tuber yield.(Table 3). A SSCP fragment originating from L-galactono-1,4-lactone dehydrogenase (*GLDH-h*) was associated with a decrease in yield of 55.7 dt/ha compared to the population mean. This marker had a high allele frequency of 81% in the population (Table 3). *HQT-f*, another SSCP fragment originating from hydroxycinnamoyl quinate CoA transferase, increased tuber yield on average by 35.9 dt/ha. Markers associated with BI, SCB, SG, TS or PM had no effect on tuber yield with the exception of *LIPIII-27-a *(Table 4). Stepwise linear regression at P < 0.001 (Table 5) resulted in the identification of three markers associated with BI, which originated from starch phosphorylase *Pho1A *(*PHO1A-b*), 4-coumarate CoA Ligase (*4CL-1b*) and the non coding SSR allele *StI013-e*. The three markers explained together 21.5% of the genetic variation of this trait. The SSR allele *StI013-a *in combination with two markers from the *Pho1B *locus (*PHO1B-1a*; *PHO1B-1b*) and the *LipIII-27-a *marker derived from a putative class III lipase explained 38.2% of the genetic variation of SG. In the case of SCB, stepwise forward regression resulted in a single significant marker derived from the candidate gene hydroxycinnamoyl quinate CoA transferase (*HQT-c*). Concerning yield, the marker fragment *GLDH-h *was the only one significant at P < 0.001. At this significance threshold no markers were identified for the traits tuber shape and plant maturity.

**Table 4:** Multiple trait associations of significant marker alleles (P < 0.01)

Marker allele	BI	SG	SCB	TS	PM	TY
*PHO1A-b*	**↑ ^1^**	**↑**	**↑**			
*PHO1A-c*	**↑**	**↑**	**↑**	**↓**		
*PHO1A-a*	**↑**		**↑**			
*PHO1B-1b*	**↓**	**↓**		**↑**		
*PHO1B-1f*	**↑**	**↑**				
*LIPIII-27-1e*	**↑**	**↑**				
*LIPIII-27-1h*	**↓**		**↓**	**↑**		
*LIPIII-27-a*		**↑**				**↓**
*POLOXA*	**↑**		**↑**			
*POLOXB*	**↓**	**↓**				
*POT32PS1-Hpy-f*	**↓**		**↓**			
*M20-b*	**↓**		**↓**			
*4Cl-1b*	**↓**	**↓**				
*4Cl-2c*	**↓**	**↓**			**↓**	
*StI024-e*	**↓**	**↓**		**↑**		
*StI013-a*	**↑**	**↑**		**↓**	**↑**	
*STM1043-b*	**↓**	**↓**				
*PHA1a-a*	**↑**			**↓**		
*CAT2-1c*	**↓**				**↓**	
*C3H-f*		**↓**		**↑**		

**^1 ^**Direction of effect, either trait value increasing (**↑**) or decreasing (**↓**)

**Table 5:** Significant marker fragments identified by stepwise linear regression (P < 0.001)

**Trait**^**1**^	BI	SG	SCB	TY
	*PHO1A-b*	*StI013-a*	*HQT-c*	*GLDH-h*
	*4CL-1b*	*PHO1B-1a*		
**Marker Fragment^2^**	*StI013-e*	*PHO1B-1b*		
		*LipIII-27-a*		
**Exp. Var.^3 ^(%)**	21,5	38,2	33,3	7,5

**^1 ^**Bruising Index (BI), Specific gravity (SG), Starch corrected bruising (SCB), Tuber yield (TY)

**^2 ^**Associated marker fragment derived from corresponding candidate gene

**^3 ^**Amount of genetic variance explained by the sum of significant markers at P < 0.001

## Discussion

This study aimed at elucidating the genetic basis of blackspot bruising in potato tubers in combination with several other important agronomical traits. The approach was to associate DNA polymorphisms at multiple functional candidate loci with natural phenotypic variation in advanced tetraploid varieties and breeding materials. We identified highly significant (P < 0.001) associations between DNA variants at candidate as well as non-candidate (SSR) loci for tuber bruising, specific gravity and yield. To the best of our knowledge, the association mapping experiment described in this paper is amongst most comprehensive and first that investigates the molecular basis of the natural variation of enzymatic browning in plants. Association genetics in model and non-model plants substantially improved the understanding of the molecular basis of various complex plant phenotypes and their impact on plant performance and economically essential traits [35,40-44]. The human genetics field provides the models how association studies influence the development of novel strategies in medicine [45-48]. In crop genetics, the combination of genomic tools with phenotypic evaluation as used in the traditional breeding process can identify molecular markers useful in "Marker-Assisted Selection" (MAS) and at the same time genetic factors that influence complex traits such as pathogen resistance and crop quality [35,38,49,50].

The experimental design (Additional file. 5) comprised the phenotypic evaluation of tuber bruising, specific gravity, yield and shape as well as plant maturity with standard methods used for variety selection at six different locations in two consecutive years. Similar to a previous association mapping experiment [35], proprietary breeding clones were evaluated at the corresponding singular breeding station, whereas a common set of potato varieties was evaluated at all six breeding stations, which facilitated joint data analysis. This experimental design makes possible to perform studies including proprietary breeding materials that otherwise would not be available, insuring that the associations found are relevant for elite germplasm. Marker-trait associations were detected using a mixed model approach, which took into account field design, environment, genotype by environment interaction, population structure and kinship. An SSR marker based genetic similarity matrix was included in the mixed model to correct for population substructure that was not detectable by principal coordinate analysis. Stepwise forward regression led to a combination of associated markers that collectively explained between 7.5 and 38.2 percent of the genetic variation of the evaluated traits.

The phenotypic data analysis during this study led to several correlations that were observed between the investigated traits. In this respect, the strong positive correlation between bruising susceptibility and specific gravity which is well known in breeding practice was confirmed. In addition, the effects of all markers associated with both traits always had the same direction. A comparable connection between tuber traits exists in the case of specific gravity and chip color, the latter being determined by the amount of reducing sugars in the tubers [35]. One explanation for the correlation between tuber bruising susceptibility and SG is close genetic linkage between different genes that control both traits independently. This might be the case for some QTL but is rather unlikely to be the case for all QTL with concerted effects on both traits that were tagged by the same marker. An alternative explanation concerning the impact of specific gravity upon bruising might be the interaction of starch grains with the surrounding internal membrane ultrastructure. Thus, a high amyloplastic load with starch grains provokes a faster and easier rupture of amyloplastic membranes upon mechanical stress. The loss of membrane integrity results on the one hand in the release of PPO and on the other hand in a mix-up of cytoplasmic and vacuolar compounds leading to the encounter of PPO with phenolic substrates [51-53]. Indeed, associations with tuber bruising and specific gravity were identified with markers derived from *PPO *genes as well as starch phosphorylase and lipase genes in concordance with this hypothesis. L-type starch phosphorylases (PHO1) are starch degrading enzymes present in plastids. The gene *PHO1A *(*Stp23*) on potato chromosome III is expressed in the amyloplasts, whereas *PHO1B *(*StpL*) on chromosome V is expressed mainly in the chloroplasts [54]. The net amount of starch in the plastids is the result of anabolic and katabolic enzymatic reactions, and carbon flux from source leaves to sink tubers. Markers originating from *PHO1A *increased mainly bruising susceptibility and to less extent specific gravity. Vice versa, markers derived from *PHO1B *were associated primarily with specific gravity and secondary with bruising. The effects of *PHO1A *and *PHO1B *allelic variants on SG verify associations found previously at these two loci in an independent association mapping experiment [35]. The observed concerted allele effects on bruising susceptibility and specific gravity also fit the hypothesis outlined above, which predicts that alleles increasing SG will indirectly increase bruising susceptibility due to a higher load of starch grains and thereby higher mechanical stress susceptibility. Sequence differences both at DNA and/or amino acid level could alter biochemical or molecular characteristics of starch phosphorylases and in consequence lead to variation in specific gravity of genotypes characterized by a given allelic composition.

Polyphenol oxidases are the major functional candidate genes for the enzymatic browning reaction. Accordingly, markers derived from at least three paralogous *PPO *genes clustered at the *PPO *locus on potato chromosome VIII were associated with tuber bruising. The markers *POLOXA *and *POT32PS1-f *were highly significantly associated with SCB, which makes them particularly interesting for MAS aiming at increased bruising resistance not compromised by low specific gravity of tubers. The association of the *POLOXA *marker with increased bruising susceptibility (BI and SCB) is especially intriguing, regarding the reported tissue specific expression of the *POTP1 *gene that is high in leaf tissue but restricted in tubers [16]. Sequencing the POLOXA amplicon revealed that the corresponding primer pair tags more than one *PPO *gene or allele (unpublished data). The observed association might therefore result from a *PPO *gene other than *POTP1*, which is expressed in tubers. Selection against the rather frequent (35%) *POLOXA *marker should improve the average bruising resistance in a breeding population. The single strand conformation polymorphism (SSCP) fragment *POT32PS1-f*, was derived from the *POT32 *gene by subjecting the CAPS marker for allele 1 described by Werij*et al*. [20] to single strand conformation analysis. *POT32 *allele 1 co-localized with a large effect QTL for enzymatic discoloration on chromosome VIII and its presence increased enzymatic discoloration [20]. The fact that SCB is virtually uncoupled from the structural components of tuber bruising makes it more comparable to enzymatic discoloration catalyzed by PPO. The *POT32PS1-f *marker decreased SCB and therefore likely represents a novel *PPO *allele that was detected due to the higher resolution of SSCP analysis and the use of tetraploid breeding material instead of the diploid mapping population employed by Werij *et al*. [20].

Furthermore, a particularly interesting candidate gene is a putative class III lipase, which has been discovered de novo by comparing the tuber proteome of bruising resistant and susceptible cultivars (Urbany et al., in preparation). SSCP markers derived from this gene showed significant associations with either BI, SCB and TS (*LIPIII-27-1h*) or BI and SG (*LIPIII-27-1e*) or SG and TY (*LIPIII-27-a*) (Table 4). The marker *LIPIII-27-1h*, present in 80% of the tetraploid individuals, was associated with reduced SCB and a trend towards round shaped tubers. A functional role of lipases in tuber bruising, specific gravity or shape is not obvious, apart from membrane modification by altering lipid composition. Recent work on the closest homolog of potato class III lipases, the *CaPLA1 *gene encoding a hot pepper phospholipase A1, points to a possible connection with tuber bruising. Seo *et al*. [55] proposed that *CaPLA1 *is involved in the regulation of cell shape and number as well as the control of carbon flux through gluconeogenesis and β-oxidation. In addition, it was postulated that the enzyme is involved in lipid signalling and thereby regulates cellular and biochemical functions in heterotrophic plant tissue. The *CaPLA1 *homologous potato unigene *LIPIII-27 *(SGN-U269327) might fulfil analogous functions in heterotrophic tuber tissue, thereby indirectly influencing tuber bruising susceptibility and specific gravity. A putative role in the regulation of cell shape and number could explain why the marker *LIPIII-27-1h *associates with tuber shape as well.

The remaining candidate markers associated with tuber bruising and specific gravity can be classified as genes involved in polyphenol synthesis (*St4Cl, Hqt, Hct, C3h, C4h*), redox homeostasis (*Cat, Zep*) and general carbohydrate and energy metabolism (*PHA1, AGPaseB*).

Populations of cultivated, tetraploid potato are characterized by large haplotype blocs, resulting from a limited number of meiotic recombination events separating the individuals and/or selection [56]. This is demonstrated by the finding of highly significant associations with SSR markers that did not encode any candidate gene. Large haplotype blocs are on the one hand favourable for the identification of diagnostic markers for breeding purposes but on the other hand prevent the verification of candidate genes by association genetics. By using linear regression for the identification of significant marker trait association, marker redundancy could be limited. The results concerning bruising validate the main candidate associations for polymorphic fragments derived from starch phosphorylase and 4-coumarate CoA ligase genes as well as the non-coding SSR locus *StI013 *previously identified by the mixed model approach (Table 5). PPO markers significantly associated with BI using mixed model statistics were also tagged by linear regression when the probability threshold was increased to P < 0.005 (data not shown). In the case of SG, markers with a significant impact on this trait by linear regression originate either from the *PHO1B *gene or the non coding SSR locus *StI013*, which also influenced BI. Regarding tuber yield and SCB, linear regression resulted in the repeated selection of significantly associated marker fragments from the L-galactono-1,4-lactone dehydrogenase and hydroxycinnamoyl CoA quinate transferase genes, respectively. These results strongly support the impact of candidate genes identified by both association analysis and linear regression on the analyzed traits. The ambiguity whether a candidate gene variant is causal for the trait effect or whether the association observed is due to LD with the causal gene has to be resolved by further functional analysis of candidate gene alleles. Candidate gene alleles associated with positive or negative trait values can be isolated and functionally characterized in heterologous model systems such as yeast, Arabidopsis or tobacco [38,57-60].

Multiple correlations between traits were evident in the investigated population. Most of these correlations can be explained on a physiological or genetic basis, as described above for tuber bruising susceptibility and specific gravity. Plant maturity was correlated with specific gravity, yield and bruising susceptibility. This might be due to a longer vegetation period (later maturity) that results consequently in higher tuber yield and specific gravity, and indirectly in higher bruising. The reason for the negative correlation between tuber shape and bruising is probably physical. The surface of round shaped tubers is more exposed to mechanical impact than oblong tubers.

The interconnection of the traits investigated and the associated marker fragments identified are a valuable resource for breeders and molecular biologists dealing with enzymatic tissue discoloration and in particular potato tuber bruising. Functional studies will have to provide further evidence on whether and how sequence diversity of candidate genes results in functional differences and thereby explains phenotypic variation. The present work is the result of a collaboration between the breeding industry and basic research and accentuates the efforts to transfer the genetic and molecular knowledge gained during such studies into the field.

## Conclusions

In conclusion, our study demonstrates that association genetics based on candidate genes in advanced breeding populations of potato allows the identification of diagnostic markers that are valuable for MAS, especially for complex traits such as tuber bruising. Twenty one highly significant (p < 0.001) and robust marker-trait associations were identified that will facilitate the diagnosis and combination of superior alleles in breeding programs. For example, selection against the rather frequent POLOXA allele and enrichment of the rare POT32 allele (*POT32PS1-Hpy-f *) associated with higher and lower bruising susceptibility, respectively, should improve tuber quality with respect to blackspot bruising.

The associations found with certain candidate genes such as lipase class III point to new structural and biochemical properties that play a role in bruising. The identified candidate genes and their associations with enzymatic discoloration and bruising are a basis to elucidate the underlying molecular networks and the sequence diversity at the given loci, not only in potato but also in other fruits and vegetables that suffer from mechanical damage.

## Methods

### Plant material

A population of 205 tetraploid potato genotypes, representing the variation for bruising susceptibility present in advanced commercial breeding materials, was used in this study. Variety names, sample coding and corresponding breeder or distributor are given in Additional file 1. The population consisted of a common set of 85 tetraploid varieties and 20 genotypes each from the current breeding programs of six breeding companies: EUROPLANT Pflanzenzucht GmbH (EU); SaKa Pflanzenzucht GbR (SA); NORIKA GmbH (NO); Bavaria-Saat GmbH (BS); Gut Bütow Dr. K. - H. Niehoff (NE); Saatzucht Berding (BE) (Additional file 5). These 120 genotypes did not contain full siblings. Historical pedigree information for most of the varieties is available at http://potatodbase.dpw.wau.nl/potatopedigree[61].

### Collection of phenotypic data

Each breeding company propagated in 2007 and 2008 the common set of 85 potato varieties and the 20 'breeder specific' clones in the field under standard phytosanitary regimes, with two replicates each at six breeding stations in Germany (EU, Kaltenberg; SA, Windeby; NO, Groß-Lüsewitz; BS, Schrobenhausen; NE, Bütow; BE, Petersgroden). Each replicate comprised 10 plants. Tuber bruising was evaluated according to the guideline from the German variety office (Bundessortenamt 30.03.2006). About 25 kg tubers were stored at 4° -6°C. Bruising tests were performed in duplicate between December (very early and early genotypes) and January (mid early to late genotypes) by exposing 8-12 kg tubers to mechanical impact in a rotating washing machine or other shaking device. The duration of mechanical stress (usually 45 - 90 sec) was calibrated against the standard varieties 'Berber', 'Tomensa', 'Laura' and 'Albatros'. The bruised tubers were stored at room temperature in the dark for 4-5 days and then cut into half. Bruising susceptibility was visually scored either as 'no discoloration', 'light discoloration' (L), 'average discoloration' (M) or 'strong discoloration' (S). The bruising index (BI) was calculated from the observations according to BI = [(0.3L + 0.5M + S)/total tuber number] × 100, with L, M and S being the fraction of tubers in the L, M or S bruising category, respectively. The bruising index ranged from 0 (resistant to bruising) to 100 (highest bruising susceptibility). Tuber shape (TS), scores ranging from 1 (completely circular) to 9 (longitudinal), and plant maturity (PM), scores ranging from 1 (very early) to 9 (very late) were also evaluated in accordance with the assessments of the German variety office. Tuber starch content (percent fresh weight) was quantified by measuring specific gravity (SG) [62]. Tuber yield (TY) was determined by tuber weight per area planted (dt/ha). The trait 'starch corrected bruising' (SCB) was obtained from the residuals of the regression of the bruising index BI on SG.

### Genotyping

Genomic DNA was isolated from freeze-dried leaf tissue as described [36]. DNA fragments were amplified by PCR (polymerase chain reaction) according to [36] using primers and annealing temperatures specified in Additional file 3. The extension time was adjusted to amplicon length ranging from 30 s to 2 min. DNA polymorphisms in the amplicons were detected by SSCP (single strand conformation polymorphism) analysis as described [36], or by agarose gel electrophoresis of amplicons with (CAPS = cleaved amplified polymorphic sequence) or without (DA = direct amplification; SCAR = sequence characterized amplified region) restriction enzyme digestion. Simple sequence repeat (SSR) markers were PCR amplified from genomic DNA of tetraploid, heterozygous individuals using primers and conditions reported in[39,63,64] the SGN database (http://solgenomics.net/) and Additional file 6. SSR alleles were separated on Spreadex gels (Elchrom Scientific, CH-6330 Cham, Switzerland) according to the supplier's instructions. DNA fragments of all marker types were recorded in each individual as 1 for presence, 0 for absence or as missing value in unclear cases.

### Phenotypic data analysis

The fact that each breeding company evaluated their clones together with the common set of 85 varieties was used to perform a joint analysis of all six locations, where each year-location combination was treated as an environment. The phenotypic data were analyzed using the statistical model:

$$y_{ijk}=\mu +g_{i}+l_{j}+{\left(gl\right)}_{ij}+r_{jk}+e_{ijk},$$

where *y_ijk _*was the entry mean for the *i*th clone in the *k*th replication of the *j*th environment, *μ *was an intercept term, *g_i _*was the genetic effect of the *i*th clone, *l_j _*was the effect of the *j*th environment, *(gl)_ij _*was the effect of genotype by environment interaction, *r_jk _*was the effect of the *k*th replicate at the *j*th location, and *e_ijk _*was the residual. For estimation of variance components, all effects were considered as random. Heritability on an entry mean basis was calculated as $h^{2}=\frac{\sigma_{g}^{2}}{\sigma_{g}^{2}+\frac{\bar{w}}{2}}$, where $\sigma_{g}^{2}$ was the genetic variance and $\bar{w}$ the mean variance of a difference between two adjusted entry means [65].

For calculation of adjusted entry means, *g_i _*was considered as fixed. Over all environments, an adjusted entry mean *M_i _*was calculated for each of the 205 clones as: $M_{i}=\overset{\wedge }{\mu }+{\overset{\wedge }{g}}_{i}$, where $\overset{\wedge }{\mu }$ and ${\overset{\wedge }{g}}_{i}$ denote the generalized least square estimates of *μ *and *g*_*i*_, respectively.

### Population structure and association analysis

Associations among the 205 tetraploid potato clones were revealed with principal coordinate analysis based on the allele frequency matrix. This analysis was performed based on 157 SSR marker alleles using the statistical software R [66].

The PK method described by [67] was used for detection of marker-phenotype associations:

$$M_{ip}=\mu +\sum P_{iu}v_{u}+a_{p}+{\overset{⌣}{g}}_{i}+e_{ip},$$

where *M_ijp _*is the adjusted entry mean of the *i*th potato clone carrying the *p*th allele, *μ *an intercept term, *v_u _*the effect of the *u*th column of the population structure matrix P, *a_p _*the effect of allele *p*, ${\overset{⌣}{g}}_{i}$ the genetic effect of the *i*th entry except for *a_p_*, *e_ip _*the residual.

Following Stich and Melchinger[68], the first *q *principal components of an allele frequency matrix were used as P matrix. *q *was chosen in such a way that the explained variance of the first *q *principal components was about 25%.

The variance of the random effects $\overset{⌣}{g}=\{{\overset{⌣}{g}}_{\text{1}},...,{\overset{⌣}{g}}_{\text{205}}\}$ and *e *= *{e*_1, 1_,..., *e*_205, p_*} *was assumed to be $\text{Var}(\overset{⌣}{g})=\text{2K}\sigma_{\overset{⌣}{g}}^{\text{2}}$ and $\text{Var}\left(e\right)=\text{R}\sigma_{r}^{\text{2}}$. K was a 205*×205 *matrix of kinship coefficients that define the degree of genetic covariance between all pairs of entries and was calculated using SPAGEDI [69]. R was a 205 × 205 matrix in which the off-diagonal elements were 0 and the diagonal elements were calculated as the square of the standard errors of the adjusted entry means. $\sigma_{\overset{⌣}{g}}^{\text{2}}$ was the genetic variance and $\sigma_{r}^{\text{2}}$ was the residual variance, both estimated by REML.

In order to take into account the LD between markers, we used in addition to a single marker approach a multiple forward regression approach to identify based on the above described PK model those marker combinations, which explain best the genotypic variation. A P-to-enter criterion of 0.001 was used.

All mixed-model calculations were performed with ASReml release 2.0 [70].

## Authors' contributions

CU and LS carried out the molecular genetics studies and the laboratory procedures. CG and CU conceived of the study, designed and evaluated the experiments and drafted the manuscript. BS carried out the statistical analysis and contributed to draft the manuscript. KHN, AB, ET, LuS, HRH, HB, HJ, JL and JS conceived of the study, provided the genetic material, carried out the phenotypic evaluations and coordinated field experiments. All authors read and approved the manuscript.

## Supplementary Material

Additional file 1 **Genotype and sample identification with the corresponding breeder/distributor affiliation**. Click here for file

Additional file 2 **Marker trait associations in the ALL population (P < 0.01)**. Click here for file

Additional file 3 **Locus and marker information**. Click here for file

Additional file 4 **Electrophoresis band distribution and identification of significant markers at P < 0.001**. In the case of SSR and DALP markers gel pictures show the band pattern and distribution of the corresponding marker fragments. Polymorphic bands are labelled as described in Additional table 3 and listed with their respective size in basepairs. SSRs were analyzed according to the procedures given in the methods section. DALP markers were separated on agarose gels. SSCP analysis was carried out as described elsewhere [36] Exemplary panes show fragment distribution and labelling. Allele ID is according to Additional table 3. The size of polymorphic SSCP fragments was not determined. Legend - (A) *StI013*; (B) *StI024*; (C) *SSR327*; (D) *POLOXA*; (E) *PHO1A*; (F) *PHO1B-1*; (G) *LIPIII-27-1*; (H) *POT32PS1-Hpy*; (I) *4CL-1*; (J); *4CL-2*; (K) *HQT*; (L); *GLDH*. Significant marker-trait associations are indicated by the specific trait abbreviation: bruising susceptibility (BI), specific gravity (SG), starch corrected bruising (SCB), and tuber yield (TY). Click here for file

Additional file 5 **Experimental design of the field experiments and data evaluation**. Breeders are listed with their corresponding abbreviation. They cultivated and evaluated the common set of 85 potato varieties in two consecutive years (2007/2008) for bruising susceptibility (BI), specific gravity (SG), tuber shape (TS), tuber yield (TY), starch corrected bruising (SCB) and plant maturity (PM). In addition each breeder evaluated 20 own breeding clones, selected based on a specific gravity below 20 percent, a balanced tuber shape, no extreme maturity phenotype, not being closely related, and one half each having a high or a low bruising phenotype. Click here for file

Additional file 6 **SSR marker information**. Click here for file
