# Supplementary material for: Association genetics in Solanum tuberosum provides new insights into potato tuber bruising and enzymatic tissue discoloration
Source: BMC Genomics. 2011 Jan 5;12:7. doi: 10.1186/1471-2164-12-7 (PMC3023753; doi:10.1186/1471-2164-12-7)
Supplement: Additional file 1 — Genotype and sample identification with the corresponding breeder/distributor affiliation. [file 1471-2164-12-7-S1.DOC]

**Additional table 1**: Plant material used for association mapping.

| **Sample** | **Genotype / variety** | **Breeder / distributer (country)** | **Sample** | **Genotype / variety** | **Breeder / distributer (country)** | **Sample** | **Genotype / variety** | **Breeder / distributer (country)** |
| --- | --- | --- | --- | --- | --- | --- | --- | --- |
| 1 | ADRETTA | Norika (D) | 36 | GUNDA | Europlant (D) | 71 | RIKEA | Saatzucht Fritz Lange (D) |
| 2 | AGAVE | Norika (D) | 37 | ILONA | SaKa Pflanzenzucht (D) | 72 | RODRIGA | SaKa Pflanzenzucht (D) |
| 3 | AGILA | Norika (D) | 38 | KARLENA | Norika (D) | 73 | ROMANO | Agrico (NL) |
| 4 | AGNES | Europlant (D) | 39 | KOLIBRI | Norika (D) | 74 | SANTANA | Van Rijn (NL) |
| 5 | AGRIA | Europlant (D) | 40 | KONDOR | Agrico (NL) | 75 | SATINA | SaKa Pflanzenzucht (D) |
| 6 | AKTIVA | Norika (D) | 41 | KRONE | Bavaria Saat (D) | 76 | SATURNA | Europlant (D) |
| 7 | ALEGRIA | Norika (D) | 42 | KUBA | Niehoff (D) | 77 | SKALA | Bavaria Saat (D) |
| 8 | ALPHA | HZPC (NL) | 43 | LADY ROSETTA | Meijer (NL) | 78 | SOLARA | Europlant (D) |
| 9 | ALWARA | Saatzucht Fritz Lange (D) | 44 | LAMBADA | Norika (D) | 79 | TALENT | Norika (D) |
| 10 | AMADO | Europlant (D) | 45 | LAURA | Europlant (D) | 80 | TOCCATA | Europlant (D) |
| 11 | AMANDA | SaKa Pflanzenzucht (D) | 46 | LOGO | Saatzucht Firlbeck (D) | 81 | TOMENSA | Europlant (D) |
| 12 | AMORA | Schaap (NL) | 47 | LOLITA | Saatzucht Firlbeck (D) | 82 | VALISA | Norika (D) |
| 13 | ANDANTE | SaKa Pflanzenzucht (D) | 48 | MARABEL | Europlant (D) | 83 | VIENNA | Saatzucht Firlbeck (D) |
| 14 | ANOSTA | Agrico (NL) | 49 | MARADONNA | Van Rijn (NL) | 84 | VITESSE | Den Hartigh (NL) |
| 15 | ARNIKA | SaKa Pflanzenzucht (D) | 50 | MARELLA | SaKa Pflanzenzucht (D) | 85 | ZAFIRA | Agrico (NL) |
| 16 | ASPIRANT | Europlant (D) | 51 | MARENA | Europlant (D) | 86-105 | B1-B20 | SaKa Pflanzenzucht (D) |
| 17 | BARAKA | HZPC (NL) | 52 | MARFONA | Agrico (NL) | 106-125 | C1-C20 | Europlant (D) |
| 18 | BELUGA | Norika (D) | 53 | MARKIES | Agrico (NL) | 126-145 | D1-D20 | Bavaria Saat (D) |
| 19 | CALLA | Europlant (D) | 54 | MATADOR | Agrico (NL) | 146-165 | E1-E20 | Saatzucht Berding (D) |
| 20 | CANDELLA | SaKa Pflanzenzucht (D) | 55 | MAXILLA | Norika (D) | 166-185 | F1-F20 | Norika (D) |
| 21 | CARMONA | SaKa Pflanzenzucht (D) | 56 | MIRAGE | SaKa Pflanzenzucht (D) | 186-205 | G1-G20 | Niehoff (D) |
| 22 | CHANTAL | Saatzucht Fritz Lange (D) | 57 | MÖWE | Norika (D) |  |  |  |
| 23 | CILENA | Europlant (D) | 58 | NATASCHA | SaKa Pflanzenzucht (D) |  |  |  |
| 24 | CINDY | Bavaria Saat (D) | 59 | NORA | Europlant (D) |  |  |  |
| 25 | DORIS | SaKa Pflanzenzucht (D) | 60 | OLGA | Europlant (D) |  |  |  |
| 26 | DRAGA | HZPC (NL) | 61 | OMEGA | Europlant (D) |  |  |  |
| 27 | ELDENA | Europlant (D) | 62 | PANDA | SaKa Pflanzenzucht (D) |  |  |  |
| 28 | ELFE | Europlant (D) | 63 | PIROL | Norika (D) |  |  |  |
| **Sample** | **Genotype / variety** | **Breeder / distributer (country)** | **Sample** | **Genotype / variety** | **Breeder / distributer (country)** |  |  |  |
| 29 | ELISABETH | Agrico (NL) | 64 | QUARTA | Europlant (D) |  |  |  |
| 30 | EXEMPLA | Saatzucht Firlbeck (D) | 65 | QUEEN | Saatzucht Firlbeck (D) |  |  |  |
| 31 | EXQUISA | Saatzucht Firlbeck (D) | 66 | RAFAELA | SaKa Pflanzenzucht (D) |  |  |  |
| 32 | FILEA | Europlant (D) | 67 | RAJA | Agrico (NL) |  |  |  |
| 33 | FITIS | Norika (D) | 68 | RANIA | Saatzucht Fritz Lange (D) |  |  |  |
| 34 | GALA | Norika (D) | 69 | REMARKA | HZPC (NL) |  |  |  |
| 35 | GRANOLA | SaKa Pflanzenzucht (D) | 70 | RENATE | Bavaria Saat (D) |  |  |  |
