# Supplementary material for: Association genetics in Solanum tuberosum provides new insights into potato tuber bruising and enzymatic tissue discoloration
Source: BMC Genomics. 2011 Jan 5;12:7. doi: 10.1186/1471-2164-12-7 (PMC3023753; doi:10.1186/1471-2164-12-7)
Supplement: Additional file 2 — Marker trait associations in the ALL population (P < 0.01). [file 1471-2164-12-7-S2.DOC]

**Additional table 2: Marker trait associations in the ALL population at P < 0.01**

| **Trait ^1^** | **Gene** | **Metabolism** | **Chromosome** | **Marker allele** | **Allele Effect ^2^** | **p value** | **Frequency [%] ^3^** |
| --- | --- | --- | --- | --- | --- | --- | --- |
| **BI** | Starch phosphorylase L-type | Starch | III | *PHO1A-b* | **↑** 17,8 ± 4 | 1.4·10^-5^ | 12.4 |
|  |  |  | III | *PHO1A-c* | **↑** 13.3 ± 3.3 | 6.3·10^-5^ | 20.8 |
|  |  |  | III | *PHO1A-a* | **↑** 20.6 ± 6 | 0.00070 | 7.1 |
|  | Starch phosphorylase L-type | Starch | V | *PHO1B-1b* | **↓** 9,1 ± 3,3 | 0.00615 | 30.6 |
|  |  |  | V | *PHO1B-1f* | **↑** 9,0 ± 3,4 | 0.00896 | 15.5 |
|  | PPO isoform potpoloxA | Polyphenols | VIII | *POLOXA* | **↑** 11.2 ± 3.1 | 0.00033 | 35.5 |
|  | PPO isoform potpoloxB | Polyphenols | VIII | *POLOXB* | **↓** 8,0 ± 3,0 | 0.00743 | 61.3 |
|  | PPO isoform POT32 | Polyphenols | VIII | *POT32PS1Hpy-f* | **↓** 14,4 ± 4,7 | 0.00228 | 10.2 |
|  |  |  | VIII | *POT32PS1Hpy-d* | **↑** 9,9 ± 3,7 | 0.00794 | 20.0 |
|  | Lipase class III | Lipids | II | *LIPIII-27-1h* | **↓** 15,8 ± 3.8 | 4.6·10^-5^ | 80.4 |
|  |  |  | II | *LIPIII-27-1e* | **↑** 9,5 ± 3,4 | 0.00536 | 21.8 |
|  | 4-coumarate CoA Ligase | Phenylpropanoids | III | *4CL-1b* | **↓** 10.6 ± 2.7 | 0.00016 | 59.1 |
|  |  |  | III | *4CL-2c* | **↓** 9.5 ± 2.8 | 0.00091 | 49.3 |
|  | Hydroxycinnamoyl transferase | Polyphenols | III | *HCT-1e* | **↑** 7,6 ± 2,9 | 0.00871 | 42.2 |
|  | ATPase; proton pump | Transport and energy | III / VI | *PHA1-A-a* | **↑** 14,4 ± 4,7 | 0.00264 | 8.8 |
|  | Catalase isoform 2 | Redox homeostasis | XII | *CAT2-1c* | **↓** 7,9 ± 3,0 | 0.00768 | 48.0 |
|  | Non coding SSR | - | II | *StI024-e* | **↓** 17.9 ± 5.1 | 0.00051 | 90.2 |
|  | Non coding SSR | - | III | *StI013-a* | **↑** 11.1 ± 3.2 | 0.00058 | 44.4 |
|  | Non coding SSR | - | III | *M20-b* | **↓** 13,5 ± 4,8 | 0.00510 | 66.2 |
|  | Non coding SSR | - | VII | *STM1043-b* | **↓** 15,0 ± 3,8 | 0.00141 | 80.9 |
|  | Non coding SSR | - | XII | *StI007-a* | **↑** 10,3 ± 3,3 | 0.00187 | 33.3 |
|  | Non coding SSR | - | XII | *StI007-c* | **↓** 9,1 ± 3,2 | 0.00438 | 36.4 |
| **Trait ^1^** | **Gene** | **Metabolism** | **Chromosome** | **Marker allele** | **Allele Effect ^2^** | **p value** | **Frequency [%] ^3^** |
| **BI** | Non coding SSR | - | V | *StI058-b* | **↓** 9,4 ± 3,4 | 0.00566 | 40.9 |
| **SG** | Starch phosphorylase L-type | Starch | V | *PHO1B-1a* | **↓** 1.20 ± 0,34 | 0.00051 | 63.1 |
|  |  |  | V | *PHO1B-1b* | **↓** 1,35 ±0,37 | 0.00036 | 30.6 |
|  |  |  | V | *PHO1B-1g* | **↑** 1.18 ± 0,39 | 0.00292 | 22.7 |
|  |  |  | V | *PHO1B-1f* | **↑** 1.31 ± 0,43 | 0.00421 | 15.5 |
|  | Starch phosphorylase L-type | Starch | III | *PHO1A-b* | **↑** 1,60 ± 0,46 | 0.00070 | 12.4 |
|  |  |  | III | *PHO1A-c* | **↑** 1.21 ± 0,38 | 0.00203 | 20.8 |
|  | PPO isoform potpoloxB | Polyphenols | VIII | *POLOXB* | **↓** 1,00 ± 0.34 | 0.00398 | 61.3 |
|  | Lipase class III | Lipids | II | *LIPIII-27-1e* | **↑** 1.22 ± 0,40 | 0.00229 | 21.3 |
|  |  |  | II | *LIPIII-27-a* | **↑** 1.25 ± 0,41 | 0.00249 | 22.6 |
|  | 4-coumarate CoA Ligase | Phenylpropanoids | III | *4CL-2c* | **↓** 0.97 ± 0,33 | 0.00328 | 49.3 |
|  |  |  | III | *4CL-1b* | **↓** 1.02 ± 0,33 | 0.00227 | 59.1 |
|  |  |  | III | *4CL-1f* | **↓** 1.02 ± 0,35 | 0.00352 | 54.2 |
|  | p-coumarate 3-hydroxylase | Phenylpropanoids | - | *C3H-f* | **↓** 2.65 ± 1,00 | 0.00914 | 7.1 |
|  | Hydroxycinnamoyl quinate CoA transferase | Polyphenols | VII | *HQT-2a* | **↓** 2.20 ± 0,68 | 0.00153 | 8.4 |
|  | ATPase; proton pump | Transport and energy | III / VI | *PHA1-A-b* | **↓** 0.94 ± 0,34 | 0.00581 | 61.3 |
|  | Glucose-1-phosphate adenylyltransferase small chain | Starch | VII | *AGPASE-c* | **↓** 1.51 ± 0,53 | 0.00584 | 24.0 |
|  | Zeaxanthin epoxidase | CCarotenoids | II | *ZEP-c* | **↓** 1.45 ± 0,53 | 0.00724 | 11.1 |
|  | Non coding SSR | - | VIII | *SSR327-a* | **↑** 1,80 ± 0,48 | 0.00026 | 17.7 |
|  | Non coding SSR | - | II | *StI024-e* | **↓** 2.06 ± 0,58 | 0.00047 | 90.2 |
|  | Non coding SSR | - | III | *StI013-a* | **↑** 1,36 ± 0,36 | 0.00023 | 44.4 |
|  | Non coding SSR | - | V | *StI058-a* | **↑** 1.06 ± 0,37 | 0.00463 | 63.1 |
|  | Non coding SSR | - | XII | *SSR20-a* | **↓** 1.16 ± 0,41 | 0.00534 | 24.9 |
| **Trait ^1^** | **Gene** | **Metabolism** | **Chromosome** | **Marker allele** | **Allele Effect ^2^** | **p value** | **Frequency [%] ^3^** |
| **SG** | Non coding SSR | - | VII | *STM1043-b* | **↓** 1.54 ± 0,55 | 0.00545 | 80.9 |
| **SCB** | Starch phosphorylase L-type | Starch | III | *PHO1A-c* | **↑** 7.6 ± 2,4 | 0.00172 | 20.8 |
|  |  |  | III | *PHO1A-b* | **↑** 9.1 ± 3,0 | 0.00244 | 12.4 |
|  |  |  | III | *PHO1A-a* | **↑** 13.0 ± 4,4 | 0.00338 | 7.1 |
|  | PPO isoform potpoloxA | Polyphenols | VIII | *POLOXA* | **↑** 7,8 ± 2.3 | 0.00064 | 35.5 |
|  | PPO isoform POT32 | Polyphenols | VIII | *POT32PS1-f* | **↓** 12.4 ± 3.4 | 0.00039 | 10.2 |
|  | PPO isoform POT32 | Polyphenols | VIII | *POT32PS1-c* | **↓** 6.8 ± 2,5 | 0.00751 | 24.4 |
|  | PPO isoform POT33 | Polyphenols | VIII | *POT33-f* | **↓** 6.6 ± 2,5 | 0.00771 | 24.9 |
|  | Lipase class III | Lipids | II | *LIPIII-27-1-h* | **↓** 10.3 ± 2.8 | 0.00028 | 80.4 |
|  | Hydroxycinnamoyl quinate CoA transferase | Polyphenols | VII | *HQT-c* | **↓** 13.0 ± 3.3 | 0.00023 |  |
|  | Cinnamic acid 4-hydroxylase | Phenylpropanoids | - | *C4H-e* | **↓** 6.5 ± 2,4 | 0.00778 | 25.8 |
|  | Non coding SSR | - | III | *M20-b* | **↓** 10.1 ± 3,5 | 0.00433 | 66.2 |
|  | Non coding SSR | - | III | *M45-d* | **↓** 6.8 ± 2,6 | 0.00896 | 25.3 |
| **TY** | L-galactono-1,4-lactone dehydrogenase | Ascorbate synthesis | X | *GLDH-h* | **↓** 55.7 ± 14.2 | 0.00012 | 81.3 |
|  | Hydroxycinnamoyl quinate CoA transferase | Polyphenols | VII | *HQT-f* | **↑** 35.9 ± 10.6 | 0.00084 | 54.7 |
|  | PPO isoform POT33 | Polyphenols | VIII | *POT33-e* | **↓** 32.3 ± 12,3 | 0.00933 | 27.1 |
|  | Lipase class III | Lipids | II | *LIPIII-27-a* | **↓** 40.9 ± 14,8 | 0.00624 | 22.6 |
|  | Pectin methyl esterase | Cell wall | - | *PEST-a* | **↓** 36.2 ± 13,8 | 0.00965 | 16.8 |
|  | Non coding SSR | - | VI | *STM0001-c* | **↓** 39.0 ± 13,1 | 0.00334 | 55.1 |
|  | Non coding SSR | - | XI | *STM0037-f* | **↑** 34.6 ± 12,7 | 0.00692 | 37.8 |
| **PM** | Starch phosphorylase L-type | Starch | V | *PHO1B-1a* | **↓** 0.44 ± 0,15 | 0.00434 | 63.1 |
|  |  |  | V | *PHO1B-b* | **↓** 0.42 ± 0,16 | 0.00780 | 48.9 |
|  |  |  |  |  |  |  |  |
| **Trait ^1^** | **Gene** | **Metabolism** | **Chromosome** | **Marker allele** | **Allele Effect ^2^** | **p value** | **Frequency [%] ^3^** |
|  | 4-coumarate CoA Ligase | Phenylpropanoids | III | *4CL-2c* | **↓** 0.44 ± 0,16 | 0.00534 | 49.3 |
|  | Hydroxycinnamoyl quinate CoA transferase | Polyphenols | VII | *HQT-1d* | **↓** 0.69 ± 0,23 | 0.00266 | 12.4 |
|  | ATPase; proton pump | Transport and energy | III / VI | *PHA1-A-1a* | **↓** 0.42 ± 0,16 | 0.00770 | 50.2 |
|  | Catalase isoform 2 | Redox homeostasis | XII | *CAT2-1c* | **↓** 0.43 ± 0,16 | 0.00907 | 48.0 |
|  | Non coding SSR | - | III | *StI013-a* | **↑** 0.54 ± 0,18 | 0.00240 | 44.4 |
| **TS** | Starch phosphorylase L-type | Starch | III | *PHO1A-c* | **↓** 0.45 ± 0,14 | 0.00225 | 20.8 |
|  | Starch phosphorylase L-type | Starch | V | *PHO1B-1b* | **↑** 0.41 ± 0,14 | 0.00467 | 30.6 |
|  | Lipase class III | Lipids | II | *LIPIII-27-1h* | **↑** 0.47 ± 0,17 | 0.00499 | 80.4 |
|  | ATPase; proton pump | Transport and energy | III / VI | *PHA1-A-a* | **↓** 0.64 ± 0,21 | 0.00201 | 8.8 |
|  | Citrate synthase | Carbohydrate | I | *CIS-c* | **↓** 0.39 ± 0,14 | 0.00664 | 24.4 |
|  | p-coumarate 3-hydroxylase | Phenylpropanoids | - | *C3H-f* | **↑** 0.91 ± 0,34 | 0.00942 | 7.1 |
|  | Non coding SSR | - | II | *StI024-e* | **↑** 0.72 ± 0,22 | 0.00141 | 90.2 |
|  | Non coding SSR | - | III | *StI013-a* | **↓** 0.37 ± 0,14 | 0.00942 | 44.4 |
|  | Non coding SSR | - | III | *M20-a* | **↑** 0.56 ± 0,22 | 0.00980 | 80.0 |

**^1^** BI: bruising index; SG: tuber starch content; SCB: starch corrected bruising; TY: tuber yield; TS: tuber shape; PM: plant maturity.

**^2^** Increasing (↑) or decreasing (↓) effect of the marker allele on the trait value compared to the population mean.

**^3^** Frequency of presence of the marker fragment in the ALL population (association of polymorphic fragments is reported if fragment frequency is above 5%)
