# Supplementary material for: Association genetics in Solanum tuberosum provides new insights into potato tuber bruising and enzymatic tissue discoloration
Source: BMC Genomics. 2011 Jan 5;12:7. doi: 10.1186/1471-2164-12-7 (PMC3023753; doi:10.1186/1471-2164-12-7)
Supplement: Additional file 3 — Locus and marker information. [file 1471-2164-12-7-S3.DOC]

**Additional table 3: Candidate genes used for association mapping**

| **Locus (accession)** | **Chr. No.** | **Encoded protein** | **Primer** | **Primer sequence**  **(5´ → 3´)**  **or reference** | **Transcript size [bp]** | **PCR product size [bp]** | **T_A_ [ °C]** | **DNA Fragments scored (assay type; enzyme) ^1^** | **Allele id.** |
| --- | --- | --- | --- | --- | --- | --- | --- | --- | --- |
| *Pot32* (U22921) | VIII | Polyphenol oxidase isoform POT32 | POT32PS1 | Werij et al. (2007) Theor. Appl. Genet. 115: 245-252 | 990 | 990 | 55 | 1 (CAPS; HphI) | a |
|  |  |  |  |  | 990 | 990 | 55 | 1 (CAPS; HpyF10IV) | a |
|  |  |  |  |  | 990 | 990 | 55 | 6 (SSCP; HphI) | Hph-a to Hph-f |
|  |  |  |  |  | 990 | 990 | 55 | 9 (SSCP; HpyF10IV) | Hpy-a to Hpy-i |
| *Pot33* (U22922) | VIII | Polyphenol oxidase isoform POT33 | POT33 | f-ctc cta tgc caa cta act ttg r-gtt acg gac ttg ttg att acg | 500 | 500 | 55 | 7 (SSCP; TaqI) | a, b, c, d, e, f, g |
| *PotP1* (M95196) | VIII | Polyphenol oxidase isoform POTPOLOXA | POLOXA | f-ctg aac caa gtc cgg gac a r-ccc gaa aca taa tca aac aca t | 1000 | 1000 | 55 | 1 (DA) | - |
| *PotP2* (M95197) | VIII | Polyphenol oxidase isoform POTPOLOXB | POLOXB | f-ctg aca ttc aac aaa g r-ttc tgt tat cgc cag ct | 200 | 200 | 52 | 1 (DA) | - |
| *StrbohA* (AB050660) | - | Respiratory burst oxidase homolog | STRBOHA | f-gga tac ggt atc ttc cgg caa g r-gct cga gct gct aaa gcc gaa tc | 485 | 485 | 55 | 4 (SSCP; MseI) | a, b, c, d |
| **Locus (accession)** | **Chr. No.** | **Encoded protein** | **Primer** | **Primer sequence**  **(5´ → 3´)**  **or reference** | **Transcript size [bp]** | **PCR product size [bp]** | **T_A_ [ °C]** | **DNA Fragments scored (assay type; enzyme) ^1^** | **Allele id.** |
| *StrbohB* (AB050661) | - | Respiratory burst oxidase homolog | STRBOHB | f-ccg cgg tga cta aag atg ag r-ccc ctc ctt ttg cta cag ag | 551 | 1000 | 55 | 12 (SSCP; MseI) | a to m |
| *StrbohC* (AB198716) | III | Respiratory burst oxidase homolog | STRBOHC | f-gga atc tac tga cat cgg aac r-ccc ttt gag ctg tgc ttt gtt ag | 558 | 700 | 55 | 6 (SSCP; MseI) | a, b, c, d, e, f |
| *St4C1-1* (M62755) | III | 4-coumarate:CoA ligase | 4CL-2 | f-gga gca att tca aca atg gc r-gca aat gcc aaa cac atc gc | 1246 | 1246 | 55 | 5 (SSCP; ApoI) | 2a, 2b, 2c, 2d, 2e |
|  |  |  | 4CL-1 | f-agg ggc atg tgg tac tgt tgt ga r-tcg ggg tgg ttg ata aga aga gc | 960 | 960 | 58 | 6 (SSCP; MseI) | 1a, 1b, 1c, 1d, 1e, 1f |
| *Hct* (SGN-U271716) | III | Hydroxycinnamoyl transferase | HCT | f-ggc tgt gga act cta atg tag r-gga gct gta gtt aac ggt att tc | 700 | 700 | 55 | 6 (SSCP; MseI) | a, b, c, d, e, f |
|  |  |  | HCT-1 | f-gct cta gtc cgc ggt gca cat aca t r-cta agc cat ccg cgg ttc caa tct a | 544 | 600 | 55 | 5 (SSCP; AluI) | Alu-a to Alu-e |
|  |  |  |  |  | 544 | 600 | 55 | 4 (SSCP; TaqI) | Taq-a to Taq-d |
| *Hqt* (SGN-U269890) | VII | Hydroxycinnamoyl CoA quinate transferase | HQT | f-gct agg gat gaa caa ggt ag r-gag gtt agg gct agc aaa gt | 860 | 800 | 55 | 6 (SSCP; MseI) | a, b, c, d, e, f |
|  |  |  | HQT-1 | f-tcc ccc acc tac cct caa ctc atc a r-ggc cta ccc cat cca aaa tca cac t | 530 | 550 | 55 | 6 (SSCP; AluI) | 1a, 1b, 1c, 1d, 1e, 1f |
| **Locus (accession)** | **Chr. No.** | **Encoded protein** | **Primer** | **Primer sequence**  **(5´ → 3´)**  **or reference** | **Transcript size [bp]** | **PCR product size [bp]** | **T_A_ [ °C]** | **DNA Fragments scored (assay type; enzyme) ^1^** | **Allele id.** |
|  |  |  | HQT-2 | f-gca cgg gac cca cca aca tct tct r-ggc cta ccc cat cca aaa tca cac t | 574 | 1000 | 55 | 4 (SSCP; AluI) | 2a, 2b, 2c, 2d |
| *C3H* (StGI-TC165348) | - | p-coumarate 3-hydroxylase | C3H | f-gaa cgc agc agg tga gat tga tg r-ata acc gcc gat ttt gac act gg | 566 | 1500 | 55 | 8 (SSCP; MseI) | a to h |
| *Dhs* (SGN-U268556) | - | 3-deoxy-7-phosphoheptulonate synthase | DHS | f-tca acc cct tct gcc ttc tcc tt r-ctc ggc agc ttt aca cca tcc tt | 575 | 1000 | 55 | 6 (SSCP; MseI) | a, b, c, d, e, f |
| *Gldh* (StGI-TC217700, | X | L-galactono-1,4-lactone dehydrogenase | GLDH | f-gaa gcc ttg cag cat gtc cga gat r-gct tgc gtc ata tgc ctg tag tgg | 560 | 4000 | 55 | 9 (SSCP; AluI) | a to i |
| *Lox* (SGN-U268109 | VIII | Lipoxygenase | LOX | f-ttt gat ggt acc cct aac ga r-atc tgg atg tgg caa act ca | 560 | 900 | 55 | 5 (SSCP; MseI) | a, b, c, d, e |
| *PHO1B = StpL* (X73684) | V | α-glucan phosphorylase L-type | PHO1B | f-gtc gca tac act aac cac act r-tgc ccg cta act aca cat a | 484 | 700 | 55 | 6 (SSCP; AluI) | a, b, c, d, e, f |
|  |  |  | PHO1B-1 | f-caa tgg cga cac taa act acc c r-aag cga ctc gtc acc tgg at | 426 | 1000 | 55 | 7 (SSCP; AluI) | 1a, 1b, 1c, 1d, 1e, 1f, 1g |
|  |  |  | PHO1B-2 | f-cgg tga tct gtt gaa ggt agt c r-tgc cca caa gga aat agt cag | 400 | 1500 | 55 | 5 (SSCP; Rsa1) | 2a, 2b, 2c, 2d, 2e |
| *PHO1A = Stp23* (D00520) | III | α-glucan phosphorylase L-type | PHO1A | f-tgc cgc atc tat tac ctc aa r-agc caa tct tca gcc acc tc | 479 | 1000 | 55 | 3 (SSCP; MseI) | a, b, c |
| **Locus (accession)** | **Chr. No.** | **Encoded protein** | **Primer** | **Primer sequence**  **(5´ → 3´)**  **or reference** | **Transcript size [bp]** | **PCR product size [bp]** | **T_A_ [ °C]** | **DNA Fragments scored (assay type; enzyme) ^1^** | **Allele id.** |
| *PHO2 =Potagpth1* (M69038) | IX | α-glucan phosphorylase H-type | PHO2-1 | f-atg ggg tta tgg ctt gag gta r-cat ccc cag ggt aga gaa cag | 400 | 900 | 55 | 4 (SSCP; MseI) | 1a, 1b, 1c, 1d, |
|  |  |  | PHO2-2 | f-gcc cag ctg cat agt gac at r-gac gcc cag aat att tag aag c | 400 | 500 | 55 | 5 (SSCP; Taq1) | 2a, 2b, 2c, 2d, 2e |
| *AGPaseB-a* | VII, XII | Glucose-1-phosphate adenylyltransferase small chain | SGN-8992**^3^** | **3** | **3** | 1500 | 55 | 3 (SSCP; MseI) | a, b, c |
| *Pha1* (X76536) | III, VI | ATPase; proton pump | PHA1-A | Chen et al (2001) Theor. Appl. Genet 102: 284-295 | 800 | 800, 1000, 1500 | 55 | 3 (DA) | a, b, c |
| *Pha1* (X76536) | III, VI | ATPase; proton pump | PHA1-A-1 | f-tcc tgg aga tgg tgt cta ctc t r-gca gta tca atg gca tcc tgg t | 560 | 900 | 55 | 5 (SSCP; TaqI) | 1a, 1b, 1c, 1d, 1e |
| *Cis* (X75082) | I | Mitochondrial citrate synthase | CIS | f-tca att ggg gaa cat cac agt r-gtc cgg cta aac cat tca aa | 700 | 200, 500, >3000 | 58 | 4 (SSCP; ApoI) | a, b, c, d |
| *Cil* (SGN-U271904) | - | ATP citrate lyase | CIL | f-tgg gga act tgg tgg acg ag r-agc cct ggc agt tac gat ggt | 600 | 300 + >3000 | 58 | 3 (SSCP; MseI) | a, b, c |
| *LipIII-25* (SGN-U269325) | - | Triacylglycerol lipase III | LIPIII-25 | f-gtg ctt tga tta tgg aat c r-atg cca tgt gat gat a | 660 | 900 | TD52 **^2^** | 1 (DA) | - |
| *LipIII-26* (SGN-U269326) | - | Triacylglycerol lipase III | LIPIII-26 | f-gag atc tcc aat tta agg c r-caa agc cac atg att tag c | 435 | 500 | 55 | 2 (SSCP; TaqI) | a, b |
| **Locus (accession)** | **Chr. No.** | **Encoded protein** | **Primer** | **Primer sequence**  **(5´ → 3´)**  **or reference** | **Transcript size [bp]** | **PCR product size [bp]** | **T_A_ [ °C]** | **DNA Fragments scored (assay type; enzyme) ^1^** | **Allele id.** |
| *LipIII-27* (SGN-U269327) | II | Triacylglycerol lipase III | LIPIII-27 | f-gtc aag gga agc atg gag taa gg r-ata ggg gga ttt tgt ggt gtc a | 650 | 1000 | 55 | 5 (SSCP; AluI) | a, b, c, d, e |
|  |  |  | LIPIII-27-1 | f-gtg atg ggg gtt tac ttc ctt tgt r-aac cat ccc att gtg ctt ctc g | 667 | 800 | 55 | 8 (SSCP; AluI) | 1a to 1h |
| *Pest2* (SGN-U270090) | - | pectin methyl esterase | PME | f-ttc ttg gcc cgg gac ata acc t r-ctc cac ggc ttg atc aaa cta acc a | 780 | 900 | 55 | 3 (SSCP; TaqI) | a, b, c |
| *Zep* (DQ206629) | II | zeaxanthin epoxidase | ZEP | f-gga ctt ccc tac aga gtt a r-cca tca tcc tca aag tct ac | 600 | 1000 | 55 | 3 (SSCP;MseI) | a, b, c |
| *C4H* (SGN-U269084) | VI? | Cinnamic acid 4-hydroxylase | C4H | f-ttc cct cct ata aat cct cac act r-agg gga tca tct tca ctc tca aat | 700 | 700 | 55 | 6 (SSCP; AluI) | a, b, c, d, e, f |
| *Cat* (AY442179) | XII | Catalase isoform | CAT | f-aga ggt ccc gtg ttg ctt gag g r-gca ggc ttt cgg gat gat gag a | 450 | 1000 | 58 | 4 (SSCP; TaqI) | a, b, c, d |
| *Cat2* (SGN-U268645) | XII | Catalase isoform 2 | CAT2-1 | f-ttt gca tac att cgc ctt ctt cta r-tcg cga tga ttg ttg tga tga c | 600 | 900 | 55 | 5 (SSCP;MseI) | 1a, 1b, 1c, 1d, 1e |

**^1^** CAPS = cleaved amplified polymorphic sequence; SSCP = single strand conformation polymorphism; DA = direct amplification. For CAPS and SSCP markers, the applied restriction enzyme is indicated in brackets

**^2^** For Touch down (TD) PCR protocols the annealing temperature was stepwise decreased starting from 60° C by 1° C until reaching the finial temperature. Afterwards standard PCR protocol was performed for additional 30 cycles.

**^3^** Marker information can be retrieved from SGN Database at Cornell (http://solgenomics.net/) by searching markers for SGN-M8992 (the corresponding marker belongs to the Conserved Ortholog Set II

(COS II).
