# Supplementary material for: Association genetics in Solanum tuberosum provides new insights into potato tuber bruising and enzymatic tissue discoloration
Source: BMC Genomics. 2011 Jan 5;12:7. doi: 10.1186/1471-2164-12-7 (PMC3023753; doi:10.1186/1471-2164-12-7)

A - *StI013* (BI / SG)

f-309
d-300
e-291
c-268
b-259
a-250
g-127

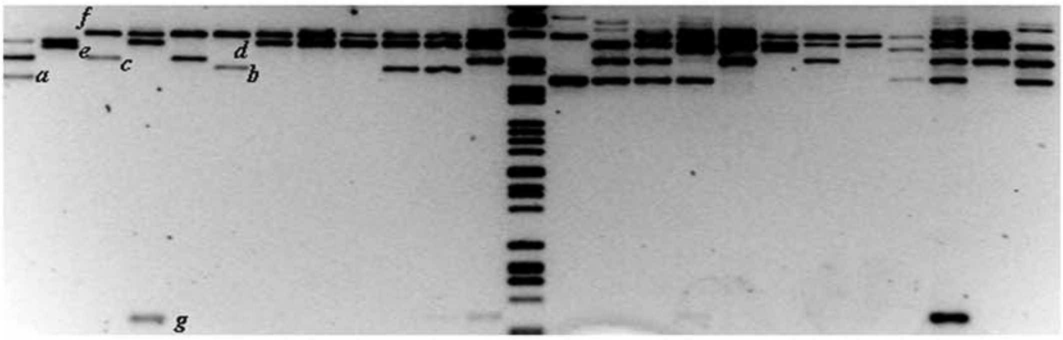

B - *StI024* (BI / SG)

e-166
d-162
h-155
c-147
b-145
g-143
a-139
f-137

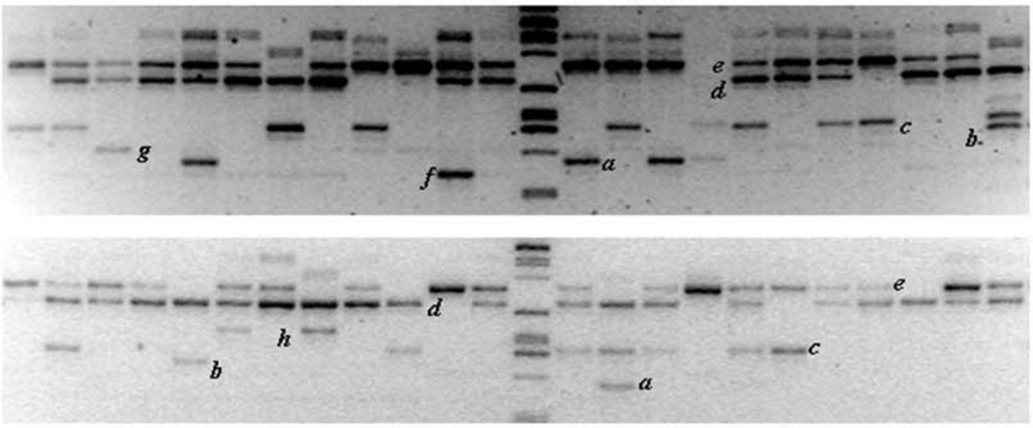

C - *SSR327* (SG)

d-480
c-347
b-341
a-291

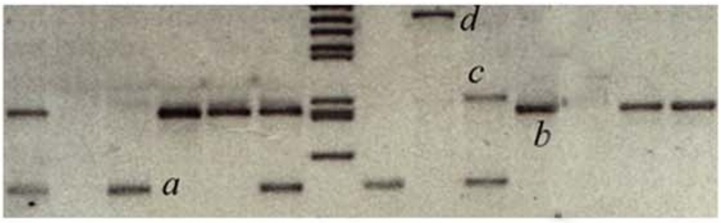

D - *POLOX A* (BI / SCB)

PoloxA-1000

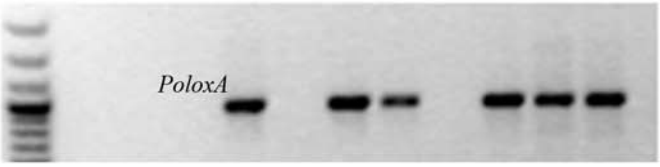

E - *PHO1A* (BI / SG)

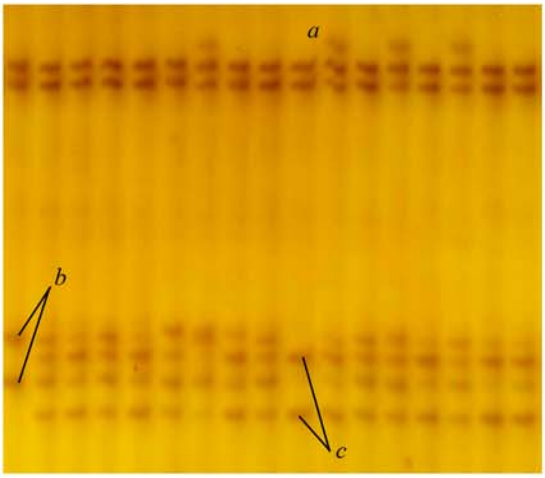

F - *PHO1B-1* (SG)

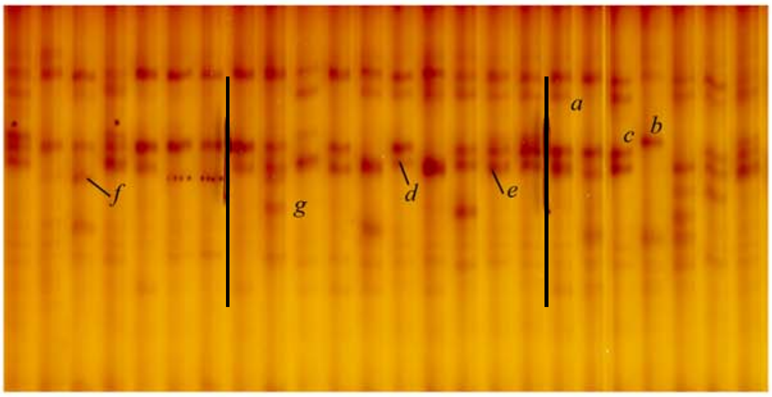

Allele ID = *1a to 1g*

G - *LIPIII-27-1* (BI / SCB)

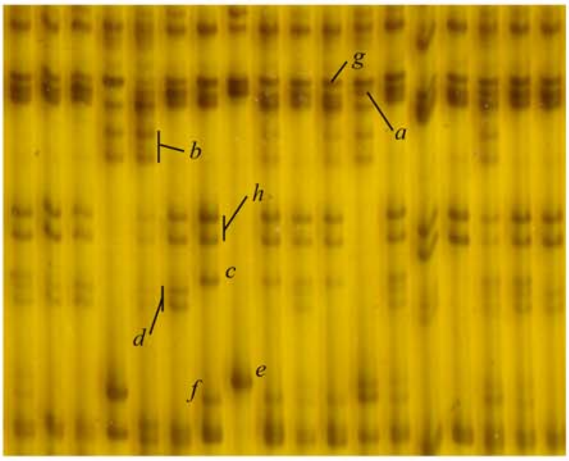

H - *POT32PS1-Hpy* (BI)

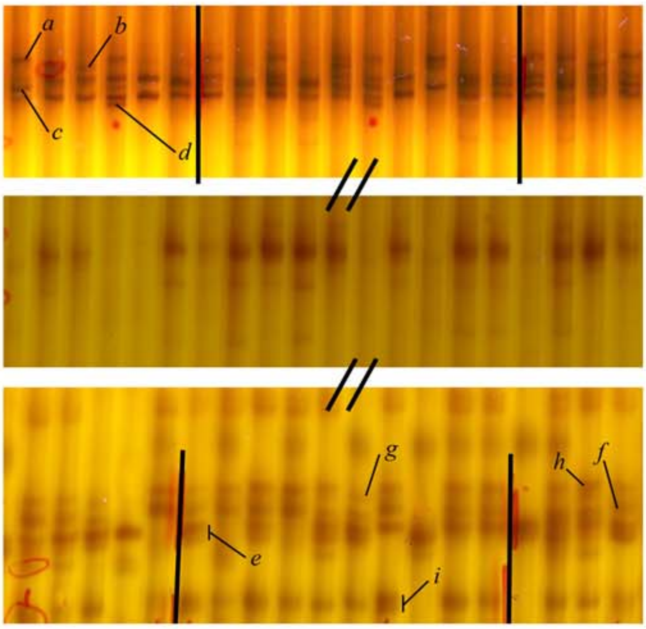

Allele ID = *Hpy-a to Hpy-i*

I - *4CL-1* (BI)

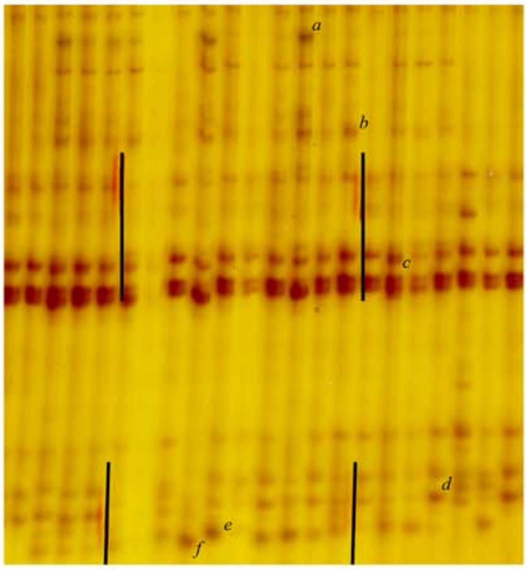

Allele ID = *1a to 1f*

J - *4CL-2* (BI)

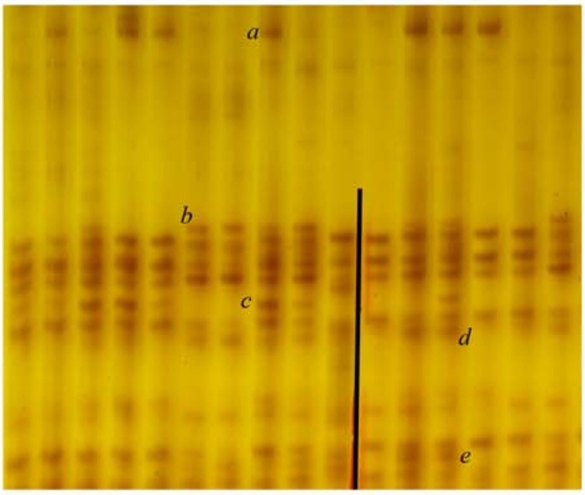

Allele ID = *2a to 2e*

K - *HQT* (SCB / TY)

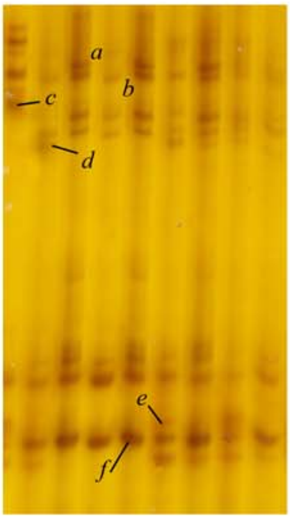

L - *GLDH* (TY)

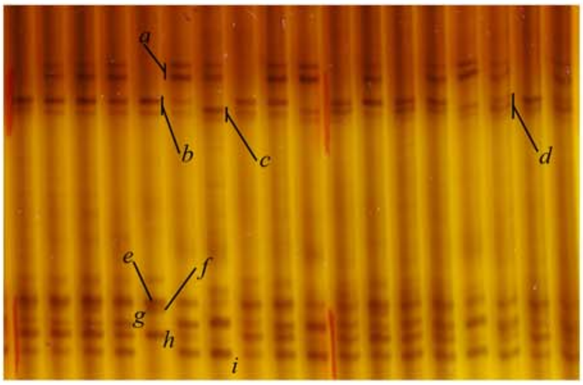

Supplement: Additional file 4 — Electrophoresis band distribution and identification of significant markers at P < 0.001. In the case of SSR and DALP markers gel pictures show the band pattern and distribution of the corresponding marker fragments. Polymorphic bands are labelled as described in Additional table 3 and listed with their respective size in basepairs. SSRs were analyzed according to the procedures given in the methods section. DALP markers were separated on agarose gels. SSCP analysis was carried out as described elsewhere [36] Exemplary panes show fragment distribution and labelling. Allele ID is according to Additional table 3. The size of polymorphic SSCP fragments was not determined. Legend - (A) StI013; (B) StI024; (C) SSR327; (D) POLOXA; (E) PHO1A; (F) PHO1B-1; (G) LIPIII-27-1; (H) POT32PS1-Hpy; (I) 4CL-1; (J); 4CL-2; (K) HQT; (L); GLDH. Significant marker-trait associations are indicated by the specific trait abbreviation: bruising susceptibility (BI), specific gravity (SG), starch corrected bruising (SCB), and tuber yield (TY). [file 1471-2164-12-7-S4.PDF]
