# Supplementary figures and images for: Association genetics in Solanum tuberosum provides new insights into potato tuber bruising and enzymatic tissue discoloration
Source: BMC Genomics. 2011 Jan 5;12:7. doi: 10.1186/1471-2164-12-7 (PMC3023753; doi:10.1186/1471-2164-12-7)

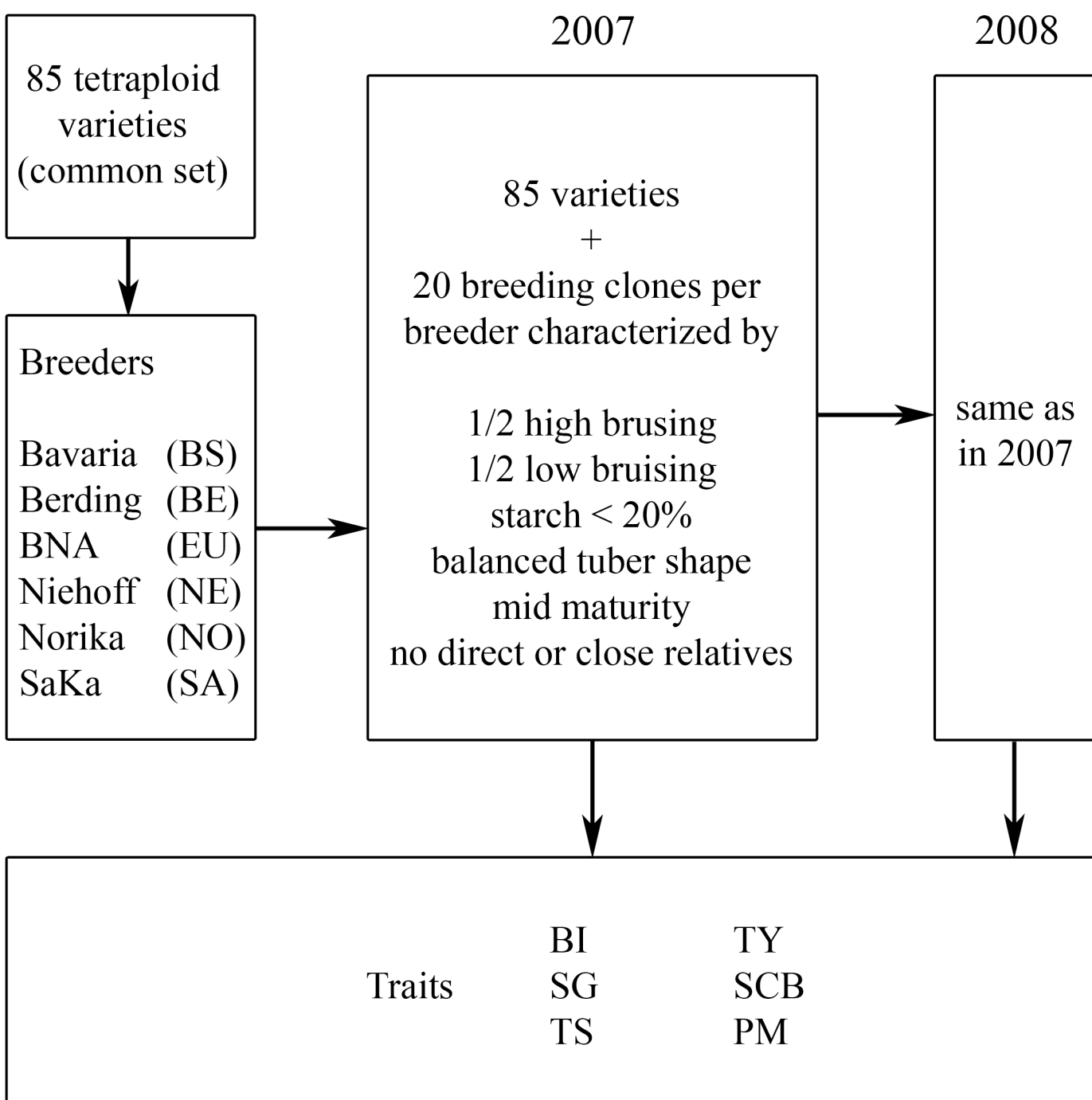

Supplement: Additional file 5 — Experimental design of the field experiments and data evaluation. Breeders are listed with their corresponding abbreviation. They cultivated and evaluated the common set of 85 potato varieties in two consecutive years (2007/2008) for bruising susceptibility (BI), specific gravity (SG), tuber shape (TS), tuber yield (TY), starch corrected bruising (SCB) and plant maturity (PM). In addition each breeder evaluated 20 own breeding clones, selected based on a specific gravity below 20 percent, a balanced tuber shape, no extreme maturity phenotype, not being closely related, and one half each having a high or a low bruising phenotype. [file 1471-2164-12-7-S5.PDF]
