# Supplementary material for: Association genetics in Solanum tuberosum provides new insights into potato tuber bruising and enzymatic tissue discoloration
Source: BMC Genomics. 2011 Jan 5;12:7. doi: 10.1186/1471-2164-12-7 (PMC3023753; doi:10.1186/1471-2164-12-7)
Supplement: Additional file 6 — SSR marker information. [file 1471-2164-12-7-S6.DOC]

**Additional table 4: SSR markers used for association mapping.**

| **Marker name** | **Encoded protein** | **Chr. No.** | **Primer** | **Reference** | **PCR product size (bp)** | **No. of DNA fragments scored** | **Annealing temperature ( °C)** |
| --- | --- | --- | --- | --- | --- | --- | --- |
|  |  |  |  |  |  |  |  |
| StI007 | weakly similar to zeatin O-glucosyltransferase | XII | StI 007 | Feingold et al. (2005) | 115-146 | 6 | TD54 ^d)^ |
| StI013 | similar to fibrillarin | III | StI013 | Feingold et al. (2005) | 240-340 | 6 | TD54 |
| StI020 | similar to bZIP transcription factor ATB2 | IV | StI020 | Feingold et al. (2005) | 106-127 | 6 | 60 |
| StI022 | similar to At4g18020 | VIII | StI022 | Feingold et al. (2005) | 114-145 | 5 | 63 |
| StI023 | weakly similar to glycine rich RNA binding protein GRP1 | X | StI023 | Feingold et al. (2005) | 160-280 | 8 | TD54 |
| StI024 | similar to nucleoporin-like protein | II | StI024 | Feingold et al. (2005) | 149-186 | 8 | TD54 |
| StI028 | homologue to YLR437CP | XI | StI028 | Feingold et al. (2005) | 170-217 | 6 | 60 |
| StI043 | non coding SSR | I | StI043 | Feingold et al. (2005) | 127-140 | 6 | TD54 |
| StI047 | MYC transcription factor | VIII | StI047 | Feingold et al. (2005) | 128-170 | 6 | TD54 |
| StI058 | homologue to TcC31.4 | V | StI058 | Feingold et al. (2005) | 77-103 | 6 | TD54 |
| STM0001 | non coding SSR | VI | STM0001 | Milbourne et al. (1998) | 113-174 | 4 | 60 |
| STM0037 | non coding SSR | XI | STM0037 | Milbourne et al. (1998) | 75-90 | 8 | 48 |
| STM0038 | non coding SSR | II | STM0038 | Milbourne et al. (1998) | 80-110 | 7 | 54 |
| STM1043 | Sucrose synthase 2 | VII | STM1043 | Milbourne et al. (1998) | 210-230 | 4 | 53 |
| STM1052 | non coding SSR | VIII | STM1052 | Milbourne et al. (1998) | 210-250 | 4 | 50 |
| STM1097 | Sucrose synthase 3 | VII | STM1097 | Milbourne et al. (1998) | 90-160 | 6 | 54 |
| STM1104 | Granule bound starch synthase | VIII | STM1104 | Milbourne et al. (1998) | 164-185 | 7 | 57 |
| **Marker name** | **Encoded protein** | **Chr. No.** | **Primer** | **Reference** | **PCR product size (bp)** | **No. of DNA fragments scored** | **Annealing temperature ( °C)** |
| STM2012 | ? | X | STM2012 | Milbourne et al. (1998) | 247-260 | 3 | 64 |
| STM3012 | ? | IX | STM3012 | Milbourne et al. (1998) | 168-213 | 4 | 57 |
| SSR308 | non coding SSR | I **^a)^** | SGN-M981 | **b)** | 310 | 1 | 55 |
| SSR20 | non coding SSR | XII **^a)^** | SGN-M693 | **b)** | 144-264 | 8 | 50 |
| SSR42 | non coding SSR | I **^a)^** | SGN-M715 | **b)** | 178-190 | 3 | 50 |
| SSR327 | non coding SSR | VIII **^a)^** | SGN-M1000 | **b)** | 291-480 | 4 | 55 |
| M4 | protease inhibitor | III | M4 | **c)** | 89-98 | 3 | TD55 |
| M16 | protease inhibitor | III | M16 | **c)** | 172-201 | 6 | TD54 |
| M17 | protease inhibitor | III | M17 | **c)** | 176-244 | 9 | TD56 |
| M20 | protease inhibitor | III | M20 | **c)** | 188-202 | 4 | TD55 |
| M27 | protease inhibitor | III | M27 | **c)** | 225-244 | 7 | TD55 |
| M45 | protease inhibitor | III | M45 | **c)** | 205-280 | 8 | TD57 |

a) Chromosomal location was deduced from mapping information available for tomato on the SGN Database at Cornell (http://solgenomics.net/)

b) The SSR markers were initially developed for tomato. Marker information can be retrieved from the SGN Database by searching markers according to name (e.g. SSR308).

c) Marker information from Odeny et al. 2010 [39]

d) For Touch down (TD) PCR protocols the annealing temperature was stepwise decreased starting from 60° C by 1° C until reaching the finial temperature. Afterwards standard PCR protocol was performed for additional 30 cycles.
